# ADVANCE integrated group intervention to address both substance use and intimate partner abuse perpetration by men in substance use treatment: a feasibility randomised controlled trial

**DOI:** 10.1186/s12889-021-11012-3

**Published:** 2021-05-25

**Authors:** Gail Gilchrist, Laura Potts, Polly Radcliffe, Gemma Halliwell, Sandi Dheensa, Juliet Henderson, Amy Johnson, Beverly Love, Elizabeth Gilchrist, Gene Feder, Steve Parrott, Jinshuo Li, Mary McMurran, Sara Kirkpatrick, Danielle Stephens-Lewis, Caroline Easton, Cassandra Berbary, Sabine Landau

**Affiliations:** 1 National Addiction Centre, Institute of Psychiatry, Psychology and Neuroscience, King’s College London, 4 Windsor Walk, London, SE5 8BB UK; 2 Department of Biostatistics and Health Informatics, Institute of Psychiatry, Psychology & Neuroscience, King’s College London, De Crespigny Park, London, SE5 8AF UK; 3 Centre for Academic Primary Care, Bristol Medical School, University of Bristol, 39 Whatley Road, Bristol, BS8 2PS UK; 4 School of Health in Social Science, University of Edinburgh, 8-9 Hope Park Square, Edinburgh, 8HQ 9NW UK; 5 Department of Health Sciences, University of York, Seebohm Rowntree Building, Heslington, York, YO10 5DD UK; 6 Independent Consulting Psychologist, Lancaster, UK; 7 RESPECT, The Green House, 244-254 Cambridge Heath Road, London, E2 9DA UK; 8 Psychological Sciences, University of Gloucestershire, The Park, Cheltenham, GL50 2RH UK; 9 Rochester Institute of Technology, 153 Lomb Memorial Drive, Rochester, NY 14623 USA

**Keywords:** Feasibility trial, Acceptability, Substance abuse treatment, Intimate partner abuse, Group intervention, Formative evaluation

## Abstract

**Background:**

Substance use is a risk factor for intimate partner abuse (IPA) perpetration. Delivering perpetrator interventions concurrently with substance use treatment shows promise.

**Methods:**

The feasibility of conducting an efficacy and cost-effectiveness trial of the ADVANCE 16-week intervention to reduce IPA by men in substance use treatment was explored. A multicentre, parallel group individually randomised controlled feasibility trial and formative evaluation was conducted. Over three temporal cycles, 104 men who had perpetrated IPA towards a female (ex) partner in the past year were randomly allocated to receive the ADVANCE intervention + substance use treatment as usual (TAU) (*n* = 54) or TAU only (*n* = 50) and assessed 16-weeks post-randomisation. Participants’ (ex) partners were offered support and 27 provided outcome data. Thirty-one staff and 12 men who attended the intervention participated in focus groups or interviews that were analysed using the framework approach. Pre-specified criteria assessed the feasibility of progression to a definitive trial: 1) ≥ 60% of eligible male participants recruited; 2) intervention acceptable to staff and male participants; 3) ≥ 70% of participants followed-up and 4) levels of substance use and 5) IPA perpetrated by men in the intervention arm did not increase from average baseline level at 16-weeks post-randomisation.

**Results:**

70.7% (104/147) of eligible men were recruited. The formative evaluation confirmed the intervention’s acceptability. Therapeutic alliance and session satisfaction were rated highly. The overall median rate of intervention session attendance (of 14 compulsory sessions) was 28.6% (range 14.3–64.3% by the third cycle). 49.0% (51/104) of men and 63.0% (17/27) of their (ex) partners were followed-up 16-weeks post-randomisation. This increased to 100% of men and women by cycle three. At follow-up, neither substance use nor IPA perpetration had worsened for men in the intervention arm.

**Conclusions:**

It was feasible to deliver the ADVANCE intervention in substance use treatment services, although it proved difficult to collect data from female (ex)partners. While some progression criteria were met, others were not, although improvements were demonstrated by the third cycle. Lessons learned will be implemented into the study design for a definitive trial of the ADVANCE intervention.

**Trial registration:**

ISRCTN79435190 prospectively registered 22nd May 2018.

**Supplementary Information:**

The online version contains supplementary material available at 10.1186/s12889-021-11012-3.

## Background

Intimate partner abuse (IPA), most commonly perpetrated by men towards women, refers to behaviour within an intimate relationship that causes harm, including physical, psychological, sexual and controlling behaviours [1]. Men with alcohol and drug use disorders have a 7- to 8-fold increase of being arrested for IPA and this risk is increased in those with a comorbid mental health disorder [2]. At least half of substance use treatment seekers have a comorbid mental health disorder [3], potentially contributing to the higher prevalence of IPA perpetration.

Unemployment, adverse childhood experiences, substance use, mental health disorders, anger, hostility, poor executive function, low empathy, relationship conflicts, misogynistic attitudes and attitudes that condone violence, and support for gender specific roles are risk factors for IPA perpetration [4–7]. The likelihood of perpetrating or experiencing physical IPA and the severity of violence increases with the number of risk factors reported [8]. For men, alcohol and drug use dependence are stronger correlates for IPA perpetration than substance use alone, suggesting that those who experience withdrawal and intoxication are at greater risk of perpetrating IPA [5]. The relationship between the psycho-pharmacological effects of substances and IPA perpetration is complex [9, 10]. Intoxication, craving, and withdrawal from substances are rarely the only explanation as IPA perpetration is *“primed and entangled with sexual jealousy, with perceptions of female impropriety and with women’s opposition to male authority”* ( [11]; pp.1).

A review of six naturalistic studies found that the prevalence of IPA was 2–3 times greater before treatment for alcohol use than after it [12]. Male to female physical assault in the year pre-treatment ranged from 60 to 71%, which fell to 19–24% in the year after treatment (4 studies). The relative risk for IPA was 2–3 times higher among patients who had relapsed following treatment (26–43% compared to 6–12%). Among men currently in treatment for substance use for varying lengths of time, 34–40% had perpetrated IPA towards a current or former female partner in the past 12-months [13, 14]. Such findings, together with the chronic, relapsing nature of substance use disorders [15], suggest that while treatment for substance use alone may reduce IPA, it may not be enough to stop IPA altogether. Given the association between substance use and IPA perpetration, programmes that address the needs of perpetrators who use substances are needed.

Evidence for the effectiveness of perpetrator interventions in healthcare settings, including substance use treatment settings, is weak. However, perpetrator interventions in health settings delivered concurrently with alcohol treatment show promise [16]. Meta-analyses of the effectiveness of different court-mandated and voluntary perpetrator programmes in reducing IPA, found significant reductions in IPA for treated men, with perpetrator programmes that address substance use and trauma being potentially more effective in reducing IPA [17]. In addition, perpetrator interventions that include motivational strategies increase attendance and reduce drop-out [18].

Despite the elevated prevalence, substance use treatment does not routinely address IPA perpetration [19, 20], with very few men in treatment for substance use being referred to perpetrator programmes [21]. Men who abuse or are dependent on alcohol or drugs are less likely to engage in perpetrator programmes [21, 22]. Few trials have considered the effectiveness of perpetrator programmes for men who use substances. A recent meta-analysis of the effectiveness of perpetrator interventions to reduce IPA by men who use substances (five of the nine trials were conducted in community (*n* = 4) or inpatient (*n* = 1) substance use treatment settings) concluded that integrated interventions targeting both IPA and substance use were not superior in reducing IPA than substance use treatment alone [23]. Physical IPA was the main outcome reported in the majority of trials, despite the perpetration of emotional abuse, coercive control and technology-facilitated abuse also being highly prevalent in this group [24, 25].

Given the high prevalence of IPA perpetrated by men in substance use treatment and lack of referral pathways; there remains a need to develop and evaluate targeted evidence-based interventions delivered in community substance use treatment settings for men who use substances and abuse their partners [26]. We developed the 16-week ADVANCE integrated intervention to target the complex ways that substance use and IPA perpetration intersect. ADVANCE is unique as it is the first intervention in substance use treatment settings that recognises IPA as involving patterns of coercive, controlling and instrumental behaviours [27]. Previous integrated interventions revert to a model of explaining only violent incidents and intoxicated violence. The ADVANCE intervention addresses these limitations by considering substance use related IPA in a manner that includes intoxicated abuse, but also recognises the role of acquisition, craving, withdrawal, and lifestyle [7–9]. We assessed the feasibility of conducting an evaluation trial of the ADVANCE intervention in substance use treatment by first exploring whether it could be done, and if so, how, including estimating the likely rates of recruitment and retention of participants, their attendance at the intervention and the acceptability of the intervention [28].

## Methods

Full details of the feasibility trial protocol are published elsewhere [29]. Ethics approval was granted by the National Health Service London - Fulham Research Ethics Committee (Reference: 18/LO/0492). The feasibility trial was prospectively registered with the ISRCTN registry (registration number ISRCTN79435190; 22/05/2018).

### Design

A multicentre, parallel group individually randomised controlled feasibility trial of the ADVANCE intervention plus substance use treatment as usual (TAU) compared to TAU only with a nested formative evaluation was conducted. Three temporal cycles of the intervention and TAU were delivered with groups of up to 18 men randomised per cycle. Cycles were run consecutively, i.e. the second cycle did not start until the first cycle had ended at each site.

### Aim

The aim of this study was to test the feasibility of conducting a trial to evaluate the ADVANCE intervention, including the acceptability of delivering the intervention in substance use treatment and the feasibility of outcome measure collection from male perpetrators and their current or ex-female partners.

### Participants and settings

Male participants were recruited by researchers from six National Health Service and voluntary sector community outpatient substance use treatment services in three regions of England (London, the West Midlands and the South West).

Researchers approached men in treatment waiting rooms or at treatment groups to introduce the study to them. Men were also identified by their keyworkers (alcohol and drug recovery workers from the substance service staff) and via recruitment flyers and posters in the service. There was a two-stage informed consent process. Men first consented to be screened for eligibility by a researcher and for the researcher to discuss their eligibility with their substance use treatment keyworker. If eligible after screening, men then consented to participate in the trial. Men were eligible if they 1) had perpetrated at least one abusive or violent behaviour towards a current or ex-female partner in the last 12 months assessed using the adapted Revised Abusive Behavior Inventory (ABI-R) [30]; 2) had face-to-face, phone, email or social media contact with their current or ex-female partner at least once in the past 12 months; 3) planned to stay in the current location for the next 6 months; 4) agreed to provide contact details of their current and/or ex-female partner/s; and 5) were able to understand and communicate in English. Men were excluded if they 1) had a current restraining order prohibiting them or anyone on their behalf from contacting their current or ex-female partner; 2) had pending court cases for IPA; 3) had pending child protection hearings or 4) were attending an intervention for IPA perpetration.

Finally, their keyworker or another staff member at the substance use service determined whether all men screened eligible were suitable to participate in the trial. This was because staff who provide the man’s treatment for substance use may be aware of additional reasons why the man may not be suitable for the study (e.g. mental or physical health problems that may interfere with participation), that the researcher was unable to collect during screening. Trial eligible men then completed a baseline interview with the researcher in the substance use treatment service.

Following randomisation, a women’s integrated support service worker contacted current or ex-female partners to offer support and more information about the trial, which researchers would provide. In the UK, accredited community perpetrator programmes must provide integrated support to (ex)partners of men taking part in such programmes. Only one current or ex-partner, corresponding to the male participant’s ABI-R responses, was contacted for each recruited male participant. Researchers telephoned current or ex-partners interested in hearing more about the trial and invited them to provide outcome data. Researchers sought the partners’ informed consent in person prior to completing the initial assessment with them. The female current or ex-partners of men participating in the trial were eligible to provide outcome data if they: 1) were aged 18 years or older; 2) had no pending court cases for IPA; 3) had no pending child protection hearings and 4) were able to understand and communicate in English. In exceptional circumstances, clinicians could override the inclusion criteria to ensure that female current or former partners were safeguarded, including: 1) where the female and male participant shared a mobile phone and 2) where the female partner lived outside the UK and therefore integrated support could not be provided. Male current or former partners and non-English speaking female current or former partners of men in the trial were not invited to provide outcome data but were offered support for their IPA victimisation.

Where possible, interviews with women took place in the substance use treatment service, the women’s support service or another service. On rare occasions, interviews with women took place in their home or a library with two researchers present.

All (non staff) participants received £5 travel expenses together with £10 cash or voucher reimbursement for completing baseline or follow-up interviews and £20 cash or voucher for taking part in focus groups or qualitative interviews.

Men and women were recruited from July 2018–April 2019. Men were followed up from December 2018–July 2019 and women were followed-up from January–July 2019.

### Randomisation and concealment

Randomisation of individual participants to substance use treatment as usual (TAU) + ADVANCE intervention (intervention arm) versus TAU only (control arm) was undertaken immediately following baseline assessment by a researcher using an online randomisation system, managed by the UK-registered King’s Clinical Trials Unit. Allocation was at the level of the individual participant, using randomly varying block sizes, stratified by a combination of sites and temporal cycles. Men and keyworkers were then informed of trial arm allocation. Researchers were not blind to treatment allocation as they were responsible for contacting men to remind them of appointments. Both statisticians were subgroup blind (only aware of coded trial arm membership) until database lock with the senior statistician remaining blind during analysis.

During a temporal cycle up to 18 consecutively recruited participants from a site were sequentially randomised. In total seven sets of up to 18 men were randomised, but the ADVANCE intervention was only delivered to six sets of men. It was not possible to deliver the ADVANCE intervention in London during cycle 1, therefore, to ensure the intervention was delivered twice in each location, three recruitment cycles were run in services in London, and two recruitment cycles were run in the West Midlands and the South West.

### Interventions

#### Control arm - usual care

Male participants in both treatment arms received substance use TAU – this included group work, individual sessions, mutual aid and opiate substitution treatment. Typically, groups are delivered weekly and individual sessions with a keyworker fortnightly, however, the number of sessions and types of TAU varies dependent on participants’ needs and available services at each site. All participants, regardless of treatment arm, were given a contact list for local services relating to substance use, mental health and IPA.

#### Intervention arm

Further details on the theory and development of the ADVANCE intervention are described elsewhere [27]. ADVANCE is a manualised evidence-informed tailored intervention developed to target IPA perpetration by men attending substance use treatment. The ADVANCE intervention focuses on developing participants’ strengths and developing healthy, non-abusive relationships. ADVANCE enhances reflective motivation, by identifying the functions of aggression, violence, and control in relationships and challenges sexist and patriarchal beliefs and attitudes. In each session, participants recognise behaviours and attitudes that need to change and learn skills for change. ADVANCE identifies the risks for IPA, including poor emotion regulation, poor stress-coping, and substance use, through education, self-regulation, and goal setting using elements from effective approaches including motivational enhancement, cognitive and dialectical behavioural therapies. Attendance was voluntary. ADVANCE comprises 2–4 individual sessions (2 compulsory) with a keyworker to set goals, develop a personal safety plan and increase motivation and readiness. This was followed by 12 weekly group sessions delivered by two trained facilitators (one female, one male) in the substance use treatment service. Participants were given out-of-session practice exercises and weekly phone check-ins with keyworkers or facilitators to address any problems arising during the intervention.

Facilitators sent weekly email feedback to participants’ keyworkers after each session to update progress, safeguarding issues and risk. Four case management meetings took place with facilitators (and keyworkers where possible) and the integrated support service workers during the intervention to manage risk. An integrated support service offered independent support for IPA victimisation to participants’ partners and also contacted the partners of men allocated to the intervention group on at least three occasions to update them on their current or ex-partner’s overall progress within the intervention and monitor risk.

Contingency management was employed to encourage attendance at the group intervention. As an incentive to achieve pro-social SMART (Specific, Measurable, Achievable, Relevant, and Time-bound) goals (e.g. going to the cinema with their children, gym attendance etc), men received a £5 voucher (for a chosen shop/service) for each of the 12 sessions attended - up to a total of £60. These were awarded at session 6 and session 12, with a £10 ‘bonus’ for attending all 12 sessions. Participants were encouraged to choose vouchers that linked with their goals, building on the ‘good lives model’ approach of the intervention [31]. Travel was reimbursed and refreshments provided.

### Feasibility parameters and acceptability assessments

#### Assessment of trial feasibility

Pre-specified criteria were used to assess the feasibility of progressing to a definitive trial: 1) ≥ 60% of eligible male participants recruited; 2) intervention acceptable to staff and male participants (attendance, focus group and qualitative interview findings; and session satisfaction ratings); 3) ≥ 70% of male and female participants followed-up at 16 weeks post-randomisation and 4) substance use and 5) IPA by men in the intervention group did not increase (average baseline level not exceeded at 16-weeks post-randomisation).

The following feasibility parameters were assessed for men: eligibility (eligible/screened), recruitment (consented/eligible), randomisation (randomised/consented) and follow-up (followed-up/randomised) rates by site and group allocation. Recruitment and follow-up rates for male participants’ current or ex-partner were also assessed. The suitability and acceptability of the proposed outcome measures was assessed with both male and female participants using the researchers’ perceptions of participants’ understanding (language and meaning of questions) and acceptability (participant refused to answer, got annoyed/frustrated or asked to end the interview) for each outcome measure using a pre-determined rating scale scored from 1 (lowest rating) to 3 (highest rating). Finally, the completeness of outcome collection was determined for each measure on both male and female participants.

#### Study measures

A number of potential outcome measures for use in a future evaluation trial were assessed at baseline (before randomisation) and after treatment (16 weeks after randomisation). A full description of all outcome measures and their scoring for the baseline assessment for men and initial assessment for women and 16 weeks post-randomisation are described in Supplementary Table S1 and in the study protocol [29]. A visit window of 4 weeks either side of 16 weeks post-randomisation was allowed for follow-up data collection.

#### Intimate partner abuse

The following measures were administered at baseline/ initial interview and 16-week follow-up to men (perpetration) and women (victimisation): the adapted Revised Abusive Behavior Inventory (ABI-R) [30], the Revised Controlling Behaviours Scale (CBS-R) [32], questions on using children against a partner [33]; technology facilitated abuse [34]; stalking [35]; or locking a partner in against their will [35]. The Communications Patterns Questionnaire-Short Form (CPQ-SF) [36] assessed perceptions of relationship conflict and communication patterns. At both baseline and follow-up, the Intimate Partner Violence Responsibility Attribution Scale (IPVRAS) [37]; the anger subscale from the Propensity for Abusiveness Scale (PAS) [38]; the Brief Self Control Scale (BSC) [39], the Balanced Inventory of Desirable Responding Short Form (BIDR-16) [40] and the University of Rhode Island Change Assessment for Domestic Violence Offenders-Revised (URICA-DV) were administered to men only [41].

#### Substance use

At baseline/ initial assessment, the Alcohol Use Disorders Identification Test (AUDIT) [42] and the Drug Use Disorders Identification Test (DUDIT) [43] were administered to men and women to assess alcohol and drug problems respectively in the past 12 months. The number of days substances were used in each of the past 4 weeks [44] and the number of days in the past 4 weeks that problems with particular substances were experienced [45] were recorded at baseline/ initial assessment and follow-up for both men and women.

#### Mental health and childhood adversities

At baseline/ initial assessment for both men and women, depression and anxiety symptoms in the past 2 weeks; and probable post-traumatic stress disorder in the past month were assessed using the Patient Health Questionnaire-9 (PHQ-9) [46], the General Anxiety Disorder-7 [47] and the Primary Care PTSD Screen for DSM-5 (PC-PTSD-5) [48] respectively. At baseline, the Standardised Assessment of Personality - Abbreviated Scale (SAPAS) was administered to men to assess possible personality disorder [49].

The Adverse Childhood Experiences (ACEs) scale was administered at baseline/ initial assessment for both men and women to assess the occurrence of 10 ACEs before the age of 18, producing an ACE score of cumulative childhood stress [50].

#### Assessment of intervention acceptability

The following process variables, collected for the intervention arm only, assessed the acceptability of the intervention: 1) the rate of intervention session attendance (number of intervention sessions attended out of the number of intervention sessions offered); 2) the number of days between randomisation and attending the first individual intervention session and 3) the number of days between randomisation and the group intervention starting. Two scales were administered at 16 weeks post-randomisation. The 12-item client version of The Working Alliance Inventory – Short Revised (WAI-SR) [51] assessed three key aspects of therapeutic alliance: agreement on the tasks of therapy, agreement on therapy goals, and affective bond development, with higher scores indicating better therapeutic alliance between client and therapist. The 12-item patient version of the California Psychotherapy Alliance Scale-Short Form (CALPAS-P) [52] measured psychotherapy alliance across 4 subscales: the patient working capacity, patient commitment, working strategy consensus, and therapist understanding and involvement. Higher scores indicated greater alliance in psychotherapy. To evaluate the group intervention content, men self-completed a brief evaluation form (Likert scale from 1 lowest - 5 highest) at the end of each of the 12 sessions.

A nested formative evaluation [53] examined experiences of delivering or attending the intervention. Focus groups or interviews with substance use treatment staff, integrated support service workers and men who attended at least one session of the intervention were conducted on completion of each cycle. Focus groups and semi-structured interviews were digitally recorded and transcribed verbatim. Data were organized and coded using NVivo by multiple coders (PR, JH, BL, GH, SD, AJ). The five steps of framework analysis were used to analyse these data: familiarisation; identifying a thematic framework; indexing; charting; and mapping and interpretation [54]. We present a summary of key findings in the Results. Men’s perspectives on motivation and change are reported elsewhere [55].

#### Assessment of fidelity of intervention delivery

All group sessions were video recorded. Study specific forms were used to rate the fidelity of the delivery of the intervention against the manualised sessions by CB, GH and SD. This information was used to revise the intervention and is not presented in this manuscript. In addition, fortnightly treatment management meetings were held with the facilitators and members of the ADVANCE team (LG, MM and SF) where any questions about the delivery could be presented and discussed.

#### Statistical analysis

As this is a feasibility study, no power calculation was carried out. Instead, the sample size target of 108 male participants (6 sets of 18 men) and 76 female current or ex partners was chosen such that all parameters required to inform the design of a definitive trial could be estimated [29].

Feasibility parameters were estimated with 95% confidence intervals (95%CI) as a measure of their precision. Confidence intervals for proportions such as the randomisation rate were generated based on an exact binomial distribution. The acceptability and understanding ratings for possible outcome measures were summarised by the median, lower quartile and upper quartile for each timepoint (baseline and 16-week follow-up). Completeness of outcome measures (number of observations and % complete) was determined for each measure at baseline and 16 weeks.

Process variables measuring the acceptability of the intervention were summarised by means and standard deviations or medians and quartiles depending on the distribution of the measure, accompanied by 95%CIs.

Potential outcome measures were summarised by arm at baseline and 16-week follow-up using appropriate descriptive statistics. Additional baseline measures were also summarised by arm to provide a description of the trial sample.

Inferential analyses estimated intervention effects in terms of potential outcome measures. These formal statistical analyses were performed on outcomes identified to be acceptable and understandable as part of the feasibility assessment and restricted to those variables that would be included as a primary or secondary outcome in a future trial. The analyses estimated the difference in mean outcomes between patients randomised to the ADVANCE intervention + TAU and TAU only by intention to treat at 16-weeks post-randomisation. Estimates of trial arm differences with associated 95%CI are presented. No formal significance tests were carried out. These effects were also standardised by dividing the estimated mean difference by the respective (pooled-group) SD at baseline. Linear regression models were used. Fixed effects included baseline measures of the outcome, trial arm (0 = control, 1 = intervention) and randomisation stratifiers, site and cycle. Baseline predictors of missingness were not included as originally planned due to the small sample size and concerns with over-parameterisation.

The number of female partners with follow-up data was too low (*n* = 17) to warrant any regression modelling and thus no statistical analyses were carried out for outcome measures obtained from female participants.

#### Economic analysis

The health economics component assessed the feasibility of conducting a cost-effectiveness analysis in a definitive randomised controlled trial. The costs of training healthcare professionals to deliver the intervention and the costs of delivering the intervention based on participants’ attendances were estimated based on team records. Self-reported use of healthcare, social care, civil services, legal and justice system contacts were first assessed for their completeness and then transformed to costs using a set of national weighted average unit costs. Health Related Quality of Life was measured using the European Quality of life 5 Dimensions-3 Level (EQ-5D-3L) [56] and the ICEpop CAPability measure for Adults (ICECAP-A) [57], the completeness of which were also assessed. The EQ-5D-3L records self-rated health on a vertical visual analogue scale from 0 to 100, where 0 is the ‘worst imaginable health state’ and 100 the ‘Best imaginable health state’ [56]. No formal cost-effectiveness analysis was conducted.

## Results

**Fig. 1:**
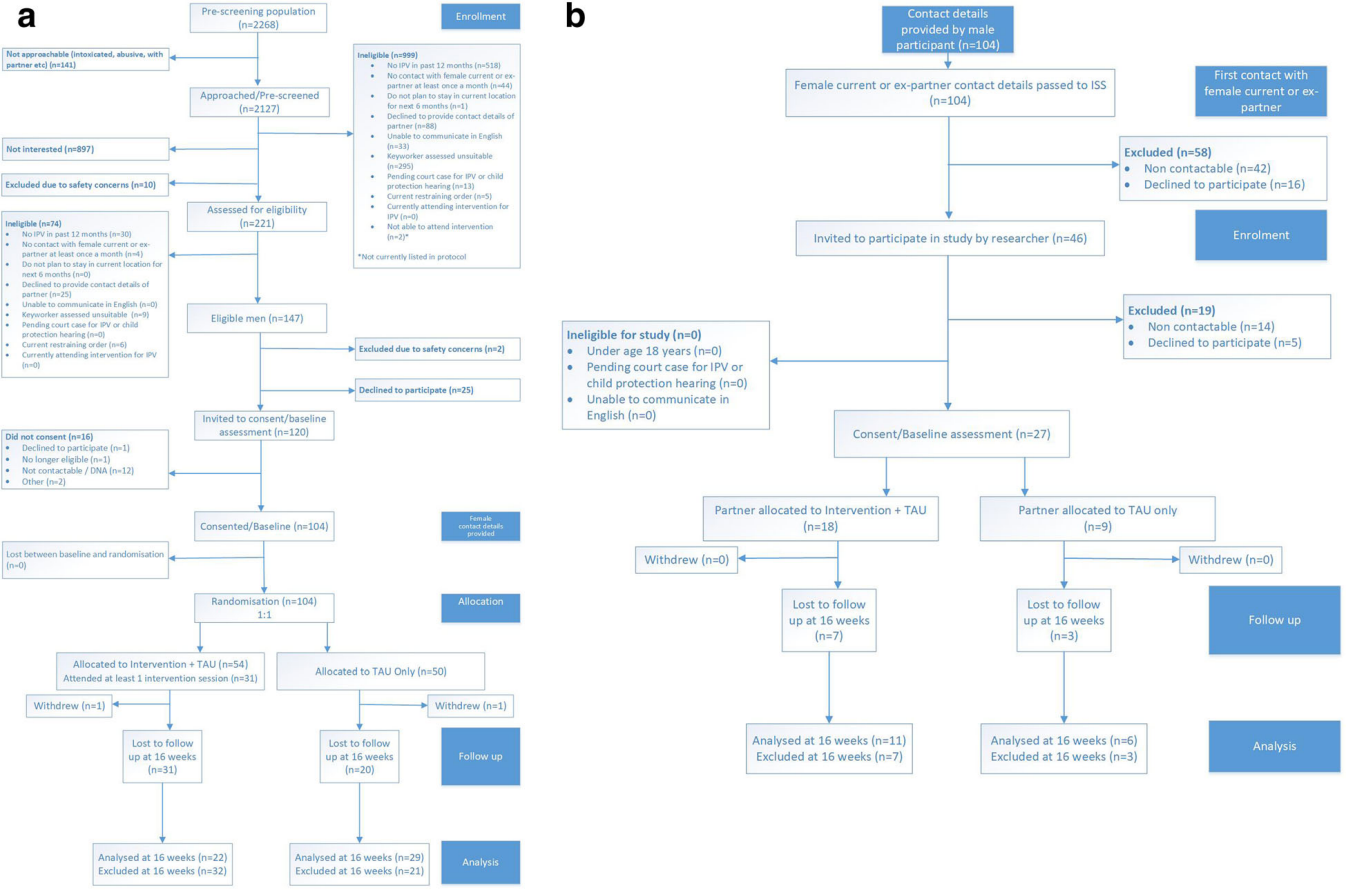
**a** CONSORT diagram for male participants in the ADVANCE feasibility trial. **b** CONSORT diagram for female current or ex-partners of male participants in the ADVANCE feasibility trial

The Consolidated Standards of Reporting Trials (CONSORT) diagram in Fig. 1a shows that although 2127 men were approached in substance use treatment waiting rooms and treatment and support groups by researchers, only 221 men (10.3% of men approached) were formally assessed for eligibility using the ABI-R by the researchers: 147 (66.5%) were eligible and 74 (33.5%) were ineligible. One hundred and four male participants were recruited and randomised to receive the ADVANCE intervention + TAU (*n* = 54) or to TAU only (*n* = 50): 39 from London, 25 from the West Midlands and 40 from the South West. Forty-nine percent of men (51/104) were followed up 16 weeks post-randomisation: 40.7% in the intervention arm and 58.0% in the control arm.

Researchers attempted to contact the 46 female current or ex-partners of male trial participants who wanted further information about the trial (Fig. 1b). Of them, 32 were contactable (69.6%) and 27 (58.7%) consented. Twenty-six percent of partners of the 104 male trial participants consented to take part in the initial assessment and 17 out of these 27 (63.0%) were followed-up at 16 weeks.

### Baseline characteristics of male trial participants and their current or ex-female partners

The baseline characteristics of male trial participants and their current or ex-partners are included in Tables 1 and 2 and Supplementary Tables S2 and S3. Male baseline characteristics were well-balanced between the trial arms. High proportions of both men and women met criteria for probable mental health disorders. Almost a third (31.7%) of men and 14.8% of partners were homeless or in temporary accommodation. Almost two thirds of men (62.5%) were in a relationship (living together or apart) and for 30.8%, although their relationship had ended and they were living apart from their ex-partner, they still had contact. Of their current or ex-partners, this was 63.0% and 11.1%, respectively. Eighty-nine percent of men (93/104) and all women reported (at least) face-to-face contact with their partner in the past 4 months at baseline. Half of the male participants and 59.3% of the female participants had children. In the past 12 months, 60.2% of men and 25.9% of women reported hazardous or harmful drinking and 48.5% and 15.4% respectively were dependent on at least one drug. Heroin (51.9%), alcohol (45.2%) and crack cocaine (39.4%) were the substances men were most commonly receiving treatment for (Table 1). Seven women were receiving treatment for substance use.

**Table 1:** Baseline measures of the male participants in the ADVANCE feasibility trial (*n* = 104) and their current or ex female partners (*n* = 27). N (%) are presented unless otherwise stated

Variable [n (%)]	Male participants			Female partners
	Trial Arm		Total (***n*** = 104)	Total (***n*** = 27)
	Intervention + TAU (***n*** = 54)	TAU only (***n*** = 50)		
**Site**				
London	20 (37.0)	19 (38.0)	39 (37.5)	17 (63.0)
West Midlands	14 (25.9)	11 (22.0)	25 (24.0)	7 (25.9)
South West	20 (37.0)	20 (40.0)	40 (38.5)	3 (11.1)
**Cycle**				
Cycle 1	22 (40.7)	21 (42.0)	43 (41.4)	11 (40.7)
Cycle 2	25 (46.3)	24 (48.0)	49 (47.1)	10 (37.0)
Cycle 3	7 (13.0)	5 (10.0)	12 (11.5)	6 (22.2)
**Age** at consent date (years) Mean (sd)	41.8 (9.7)	42.4 (10.5)	42.1 (10.1)	41.8 (12.1)
**Ethnic group**				
White	37 (69.8)	41 (82.0)	78 (75.7)	16 (59.3)
Black	8 (15.1)	3 (6.0)	11 (10.7)	3 (11.1)
Asian	5 (9.4)	5 (10.0)	10 (9.7)	5 (18.5)
Other	3 (5.7)	1 (2.0)	4 (3.9)	3 (11.1)
**Level of education**				
No formal qualifications	7 (12.9)	10 (20.0)	17 (16.4)	4 (14.8)
Secondary (GCSE’S, A levels or equivalent)	32 (59.3)	22 (44.0)	54 (51.9)	15 (55.6)
Higher/further (college or university)	9 (16.7)	12 (24.0)	21 (20.2)	7 (25.9)
Other qualifications	6 (11.1)	6 (12.0)	12 (11.5)	1 (3.7)
**Employment status**				
Employed	9 (16.7)	9 (18.0)	18 (17.3)	9 (33.3)
Looking after your home/family	1 (1.9)	2 (4.0)	3 (2.9)	5 (18.5)
Unemployed or receiving sickness benefits	38 (70.4)	33 (66.0)	71 (68.3)	8 (29.6)
Retired from paid work	2 (3.7)	2 (4.0)	4 (3.8)	2 (7.4)
Other	4 (7.4)	4 (8.0)	8 (7.7)	3 (11.1)
**Relationship status**				
Together and living together	21 (38.9)	19 (38.0)	40 (38.5)	15 (55.6)
Together but living apart	12 (22.2)	13 (26.0)	25 (24.0)	2 (7.4)
In the process of splitting up	1 (1.9)	2 (4.0)	3 (2.9)	2 (7.4)
The relationship has ended and we are living apart with no contact	1 (1.9)	2 (4.0)	3 (2.9)	1 (3.7)
The relationship has ended and we are living apart and still have contact	18 (33.3)	14 (28.0)	32 (30.8)	3 (11.1)
Something else	1 (1.9)	–	1 (1.0)	4 (14.8)
**Living arrangements**				
Homeless or in temporary accommodation	19 (35.2)	14 (28.0)	33 (31.7)	4 (14.8)
Housed - in own tenancy	20 (37.0)	24 (48.0)	44 (42.3)	14 (51.9)
Housed - in someone else’s tenancy	12 (22.2)	10 (20.0)	22 (21.2)	4 (14.8)
Other	3 (5.6)	2 (4.0)	5 (4.8)	5 (18.5)
**Has children**	30 (55.6)	22 (44.0)	52 (50.0)	16 (59.3)
**Hazardous and harmful alcohol use** in past 12 months (AUDIT scale)	30 (56.6)	32 (64.0)	62 (60.2)	7 (25.9)
**Highly probable dependent on one or more drugs** in past 12 months (DUDIT scale)	28 (51.9)	22 (44.9)	50 (48.5)	4 (15.4)
**Receiving treatment for:**				
Heroin	28 (51.9)	26 (52.0)	54 (51.9)	5 (18.5)
Cocaine	6 (11.1)	5 (10.0)	11 (10.6)	0 (0.0)
Crack	19 (35.2)	22 (44.0)	41 (39.4)	3 (11.1)
Cannabis	4 (7.4)	2 (4.0)	6 (5.8)	0 (0.0)
Alcohol	22 (40.7)	25 (50.0)	47 (45.2)	2 (7.4)
**Substance use treatment episode length** (if in treatment)				
< 6 months	25 (46.3)	22 (44.0)	47 (45.2)	1 (14.3)
6–12 months	9 (16.7)	13 (26.0)	22 (21.2)	1 (14.3)
> 12 months	20 (37.0)	15 (30.0)	35 (33.7)	5 (71.4)
**Screened positive for personality disorder** (SAPAS scale)	47 (87.0)	41 (83.7)	88 (85.4)	20 (74.1)
**Adverse Childhood Experiences** Total Score - mean (sd)	4.5 (2.4)	4.5 (2.5)	4.5 (2.4)	3.3 (2.7)

**Table 2:** Outcome measures of the male participants in the ADVANCE feasibility trial (*n* = 104)

Variable			Baseline			16 weeks follow-up		
			Intervention + TAU (***n*** = 54)	TAU only (***n*** = 50)	Total (***n*** = 104)	Intervention + TAU (***n*** = 22)	TAU only (***n*** = 29)	Total (***n*** = 51)
**Mental Health**	PHQ-9 Total Score *Scale Range 0–27*	N	54	50	104	22	29	51
		mean (sd)	12.5 (6.9)	12.5 (5.7)	12.5 (6.3)	10.0 (6.2)	10.9 (6.0)	10.5 (6.0)
	PHQ-9 score 10 or more	n (%)	31 (57.4)	34 (68.0)	65 (62.5)	10 (45.5)	16 (55.2)	26 (51.0)
	GAD-7 Total Score *Scale Range 0–21*	N	54	50	104	22	29	51
		mean (sd)	10.3 (6.5)	10.3 (5.9)	10.3 (6.2)	7.9 (6.0)	9.0 (5.3)	8.5 (5.5)
	GAD-7 score 10 or more	n (%)	24 (44.4)	25 (50.0)	49 (47.1)	7 (31.8)	12 (41.4)	19 (37.3)
	Primary Care PTSD Screen Total Score *Scale Range 0–5*	N	52	50	102	22	29	51
		median (LQ-UQ)	4.0 (1.0–6.0)	4.0 (0.0–5.0)	4.0 (1.0–5.0)	2.5 (1.0–5.0)	3.0 (1.0–5.0)	3.0 (1.0–5.0)
	PTSD Screen score 3 or more	n (%)	32 (61.5)	30 (60.0)	62 (60.8)	11 (50.0)	15 (51.7)	26 (51.0)
**Intimate Partner Abuse in the past 4 months**	ABI-R Perpetration Total Score *Scale Range 25–125*	N	54	50	104	22	28	50
		median (LQ-UQ)	33.0 (29.0–39.0)	34.5 (28.0–38.0)	33.0 (29.0–39.0)	29.0 (26.0–31.0)	30.0 (28.5–33.5)	29.5 (27.0–32.0)
	ABI-R Perpetration reported (> 1) on any item	N	54	50	104	22	28	50
		n (%)	50 (92.6)	44 (88.0)	94 (90.4)	17 (77.3)	25 (89.3)	42 (84.0)
	ABI-R Perpetration Physical Score *Scale Range 9–45*	N	54	50	104	22	28	50
		median (LQ-UQ)	9.5 (9.0–11.0)	10.0 (9.0–11.0)	10.0 (9.0–11.0)	9.0 (9.0–9.0)	9.0 (9.0–10.0)	9.0 (9.0–9.0)
	ABI-R Perpetration Psychological Score *Scale Range 13–65*	N	54	50	104	22	28	50
		median (LQ-UQ)	20.0 (17.0–24.0)	20.5 (15.0–26.0)	20.0 (16.5–24.5)	17.0 (14.0–19.0)	17.5 (16.0–20.5)	17.0 (15.0–20.0)
	ABI-R Perpetration Sexual Score *Scale Range 3–15*	N	54	50	104	22	28	50
		median (LQ-UQ)	3.0 (3.0–3.0)	3.0 (3.0–3.0)	3.0 (3.0–3.0)	3.0 (3.0–3.0)	3.0 (3.0–3.0)	3.0 (3.0–3.0)
	Controlling Behaviours Scale (Partial) Perpetration Total Score Scale Range 0–16	N	54	50	104	22	28	50
		median (LQ-UQ)	1.0 (0.0–2.0)	1.0 (0.0–2.0)	1.0 (0.0–2.0)	0.0 (0.0–2.0)	0.0 (0.0–2.0)	0.0 (0.0–2.0)
	Propensity for Abusiveness Scale (Anger subscale) total score *Scale Range 12–60*	N	53	50	103	21	28	49
		mean (sd)	36.4 (10.4)	36.1 (9.8)	36.3 (10.1)	33.1 (12.7)	32.6 (11.8)	32.8 (12.1)
**Self-management**	Brief self-control scale total score *Scale Range 13–65*	N	54	50	104	21	29	50
		mean (sd)	33.0 (9.1)	34.0 (7.5)	33.5 (8.3)	34.4 (9.8)	35.3 (9.2)	34.9 (9.4)
**Desirable responding**	BIDR-16 total score *Scale Range 16–128*	N	54	50	104	22	29	51
		mean (sd)	66.7 (13.9)	71.2 (16.0)	68.9 (15.0)	67.3 (17.4)	70.4 (15.1)	69.1 (16.0)

*LQ* lower quartile, *UQ* upper quartile, *sd* standard deviation

### Feasibility parameters

Feasibility parameters for male participants and their current or ex-female partners are detailed in Table 3. Only about 7% of men approached were eligible for the trial (eligibility rate); of men screened, 66.5% were eligible. Approximately 71% of eligible men consented to take part in the trial (consent rate), and all men who consented to take part in the trial were randomised (randomisation rate). Only about half, 49%, of randomised men were successfully followed up (follow-up rate), but this improved by cycle 3: 55.8% (24/43) of men were followed-up in cycle 1, 30.6% (15/49) in cycle 2 and 100% (12/12) in cycle 3. Female partner recruitment to the study was more difficult than anticipated: only 26% of female partners consented to participation, and 63% of those were followed-up.

**Table 3:** Feasibility parameter estimates and 95% confidence intervals

Male participants			Female current or ex-partners of men in the trial		
Feasibility parameters	Proportion	Proportion % [95%CI]	Feasibility parameters	Proportion	Proportion % [95%CI]
Eligibility rate (eligible men/ approached men)	147/2127	6.9 [5.9, 8.1]	–	–	–
Recruitment rate (consented men/eligible men)	104/147	70.7 [62.7, 78.0]	Recruitment rate (consented women/ men randomised)	27/104	26.0 [17.9, 35.5]
Randomisation rate (randomised men/consented men)	104/104	100.0 [96.5, 100.0^a^]	–	–	–
Follow-up rate 16 weeks post-randomisation (followed-up men/ randomised men)	51/104	49.0 [39.1, 59.0]	Follow-up rate 16 weeks post-randomisation of male participant (followed-up women/ consented women)	17/27	63.0 [42.4, 80.6]

^a^1 sided 97.5% confidence interval

Acceptability, understanding and completeness of outcome measures are reported in Table S1. All measures have a very high acceptability and understanding rating (median 3.0), apart from the acceptability of the IPVRAS and the URICA-DV (median 2.0 and 1.0 respectively).

### Potential patient-centred outcome measures

Measures on the male participants that had low understanding and low acceptability rating (URICA-DV, IPVRAS), low completeness (URICA-DV, IPVRAS), poor distributional properties (substance use variables were bimodal), unvalidated scales (other questions), and also any victimisation measures were not considered as potential outcomes for a future trial. Descriptive statistics at baseline and 16-weeks follow-up for the potential outcomes of the male participants are displayed in Table 2, reported by trial arm and overall and for female participants in Supplementary Table S3.

**Fig. 2:**
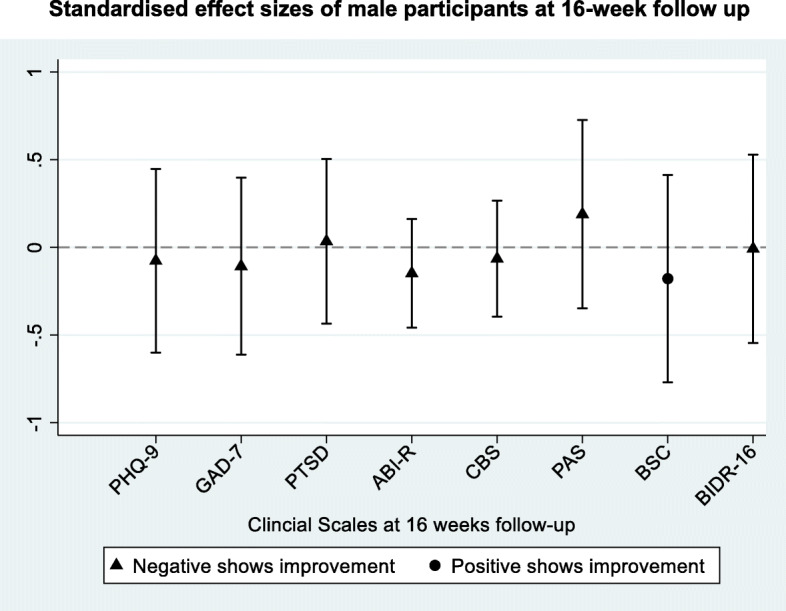
Standardised effect sizes of male participants at 16-week follow up

Estimated treatment differences for male participants at follow-up on the eight potential outcome measures for a future trial were calculated and are presented in Supplementary Table S4. Fifty-one male participants were followed up at 16 weeks post-randomisation. Figure 2 illustrates these estimated differences after standardising them so that all effects are expressed in units of baseline standard deviations. The sign of the estimated group difference indicated improvement on all scales, except for the Primary Care PTSD Screen (PC-PTSD-5), the anger subscale from the Propensity for Abusiveness Scale, and the Brief Self-Control Scale. Standardised estimated effect sizes are in the small range (< 0.2) and all associated confidence intervals cross the line of no difference, zero.

### Intervention acceptability

The ADVANCE intervention was delivered to six sets of men. It was not always possible to recruit 18 men as planned to each set. Fifty-four men were randomised to the intervention arm, but the intervention was only available to 47 men. It was not possible to deliver the ADVANCE intervention in London during cycle 1 due to the delay between randomisation and setting a start date for the group intervention resulting in five of the six men allocated to the intervention group being no longer available (due to employment, relocation or change in life circumstances) (*n* = 3) or no longer interested (*n* = 2). Therefore, a third cycle was undertaken in a new treatment service in London.

Table 4 describes the process variables used to assess the acceptability of the ADVANCE intervention for men who were offered the intervention. The mean number of days from randomisation to the first individual session for those men who attended the first individual session (*n* = 24) was 35.4 days but reduced from cycle 1 (46.7 days) to cycle 3 (17.2 days) by almost 30 days. There was a considerable delay (mean 42 days) between participants being randomised and the group intervention starting, with large variability between participants. This was due to the sequential randomisation design of the trial. The mean number of days from randomisation to the group intervention starting, regardless of whether men attended the intervention, reduced from cycle 1 (46.6 days) to cycle 3 (39.1 days) by 7.5 days. Sixty-six percent (31/47) attended at least one of the 14 compulsory sessions. The overall median rate (28.6%) of intervention session attendance was low (of 14 compulsory sessions only): 35.7% in cycle 1, 14.3% in cycle 2, increasing to 64.3% by cycle 3.

**Table 4:** Process variables on the male participants offered the ADVANCE intervention (*n* = 47)

Process Variable	n	Mean (sd) / median (LQ-UQ)	95% CI	Range
**Compliance (out of 14 compulsory sessions)**				
Rate of intervention session attendance (%) – median (LQ-UQ) *100% = 14 sessions*	47	28.6 (0.0–50.0)	7.1–35.7	0–92.9
Cycle 1	15	35.7 (0.0–50.0)	7.6–63.8	0–92.9
Cycle 2	25	14.3 (0.0–35.7)	−2.7-31.3	0–92.9
Cycle 3	7	64.3 (42.9–78.6)	44.0–84.5	0–85.7
**California Psychotherapy Alliance Scales – Patient Version Short Form (CALPAS-P SF) at 16 weeks follow-up**				
CALPAS-P SF Score – mean (sd) *Range 1–7*	17	5.9 (0.9)	5.4–6.4	3–7
Understanding Rating of the CALPAS-P SF – median (LQ-UQ)	17	3.0 (3.0–3.0)	3.0–3.0	1–3
Acceptability Rating of the CALPAS-P SF – median (LQ-UQ)	17	3.0 (3.0–3.0)	3.0–3.0	2–3
**Working Alliance Inventory – Short Revised (WAI-SR) at 16 weeks follow-up**				
WAI-SR Total Score – mean (sd) *Range 12–60*	16	48.8 (8.8)	44.1–53.5	34–60
Understanding rating of the WAI-SR – median (LQ-UQ)	17	3.0 (3.0–3.0)	3.0–3.0	1–3
Acceptability rating of the WAI-SR – median (LQ-UQ)	17	3.0 (3.0–3.0)	3.0–3.0	2–3
**Time between randomisation and intervention**				
Time between randomisation and first individual intervention session (preparation session A), for those attending session A only (days) – mean (sd)	24	35.4 (25.0)	24.8–45.9	0–82
Cycle 1	7	46.7 (21.1)	27.2–66.2	9–73
Cycle 2	12	36.3 (27.1)	19.1–53.5	6–82
Cycle 3	5	17.2 (16.3)	−3.0-37.4	0–43
Time between randomisation and group intervention starting (days) – mean (sd)	47	42.3 (23.2)	35.5–49.1	2–89
Cycle 1	15	46.6 (21.1)	34.9–58.3	2–74
Cycle 2	25	40.6 (27.1)	29.4–51.8	6–89
Cycle 3	7	39.1 (8.9)	30.9–47.4	29–51

*LQ* lower quartile, *UQ* upper quartile, *sd* standard deviation

Reasons for session non-attendance were participant no longer continuing with the study (*n* = 163 sessions), participant decided not to attend (*n* = 33 sessions), participant was ill (*n* = 23 sessions) or participant was attending other appointments or was in hospital or rehabilitation (*n* = 16 sessions). Men who did not attend any intervention sessions were younger (mean age: 39 vs 44 years). A higher proportion of men who did not attend any intervention sessions also lived in homeless or temporary accommodation (37.5% vs 25.8%); suffered from moderate-severe depression (68.8% vs 51.6%), anxiety (56.3% vs 38.7%) or post-traumatic stress disorder (75.0% vs 58.6%) measured using the PHQ-9, GAD-7 and PC-PTSD-5.

Participant-reported therapeutic alliance was high, with a mean CALPAS-P score of 5.9 (highest therapeutic alliance score = 7) and a mean WAI-SR score of 48.8 (highest therapeutic alliance score = 60). An evaluation-rating scale designed for the purpose of evaluating the intervention sessions was completed at the end of each group session. The lowest score was 1 (poor rating) and the highest was 5 (high rating) for each item. Men rated that they understood the purpose of each group session (mean scores 4.19–4.82 across sessions), found the exercises relevant and informative (mean scores 4.00–4.73 across sessions) and rated the sessions highly (mean scores 4.13–4.91 across sessions).

As part of the formative evaluation of the acceptability of the intervention, seven focus groups and seven interviews with 31 facilitators and substance use keyworkers (cycles 1–3), seven interviews with men (cycle 1) allocated to the intervention arm, and a focus group with five men who attended at least one session of the intervention (cycle 3) were conducted. Additional interviews were conducted with men in the control arm. More detailed findings about perspectives on motivation and change in IPA behaviours from the formative evaluation are published elsewhere [48].

Across all cycles, keyworkers and intervention facilitators felt ADVANCE had the potential to be easily integrated into their services. They felt ADVANCE was unique and much needed since no other intervention addressed IPA in substance use treatment services. They supported the intervention model and aim of encouraging clients to think about behaviour change in relation to sensitive and difficult issues. Recruiting men who were ready and motivated to deal with their substance use and IPA was deemed important. Incentives were useful although staff considered these were not the only motivation for men attending. They argued that using de-stigmatising language such as ‘improving relationships’ rather than IPA may have encouraged men to participate in the trial.

Facilitators required protected time to prepare for intervention delivery: *‘my manager has been really flexible and said, “You just need to feel comfortable and if that takes all of Tuesday, then you’ve got it”, but that’s not really a sustainable model going forward’* (Facilitator, South West cycle 1), otherwise it impacted on their existing workload: *‘Then any notes that you’d left, you had to do them the next day because by the time we’d done that, we were straight into the session, finished at 7:00 and locked*’ (Facilitator, West Midlands cycle 1). The fortnightly integrity support from the ADVANCE team helped clarify any issues about session content: *‘once I’d spoken to [ADVANCE integrity support] a few times and I understood it, I realised how much it flowed [ …*] *If I’d had more discussions with her earlier on, I think that would have developed earlier and I would have had a better understanding of some things’* (Facilitator, London cycle 3). Safeguarding procedures, such as the four required case management meetings and weekly follow-up with the integrated support workers, had worked well in managing and mitigating risk. Staff recognised the importance of multi-agency working: ‘*We had one incident of risk and we referred to social services. We just worked together and spoke to each other. It worked okay. Working with other agencies is definitely part of what I do, and I think it’s brilliant working with other agencies. I think it’s the best thing that you can do’* (Facilitator, London Cycle 3) *‘I did come to those meetings [with integrated support services] I think it’s absolutely crucial for it to be there*’ (Facilitator, Cycle 2, South West).

Men attending the intervention were motivated to improve their relationships: *‘being in a new relationship, I wanted to address some issues that I had’* (P030032)*,* ‘*Well, I just wanted, because I had assaulted her, I just wanted it to try and make myself better. I just wanted to get out of trouble, initially, but then, as time went on, I enjoyed it’* (P020041) and to address their substance use **‘***I wanted to find a way to improve myself and to make me more of a happier person by not using‘*(P010100).

Men found the intervention relevant: *‘I’ve learnt things from this course which I can put into practice****.’*** (P010099), ‘*That crisis plan, yes... it was a very good thing to learn, for me personally*’ (P020041). One facilitator reported that ‘*we did exactly what it said in the manual and we let everybody go round the table and say two last words. One of them, his words were ‘life changing”’* (Facilitator, London Cycle 3).

Across the cycles, men benefited from being in a group of men with shared experiences: *‘when I saw the other guys, I thought it would be okay ... Everyone was in the same sort of boat … so it was easier to deal with.’* (P030032) that despite ‘*fragmented … attendance’* men *‘were able to support each other’* when they *‘were there together’* (P020017). Similarly, men appreciated the support they received from other group members.

In all three cycles men were impressed by the skills, enthusiasm and sensitivity of the facilitators: *‘I found them really friendly and welcoming. So that makes a lot of difference, actually, who’s taking the group. Obviously, they’re not judgemental and they listen, so yes, I think they’ve done a really good job’* (P030031)*.* The rapport developed between facilitators and participants motivated men to continue attending the group: *‘I did actually look forward to going … partly, that was because of them’* (P030013).

#### Intervention safety

Two serious adverse events were recorded. Following a review by Data Management and Ethics Committee and the NHS Ethics Committee, these were assessed as not related to the trial.

#### Progression to a full trial

Five pre-specified criteria were used to assess the feasibility of conducting an evaluation trial of the ADVANCE intervention in substance use treatment. *1) The intervention was acceptable to the majority of staff and male participants.* The formative evaluation supports the acceptability of the intervention to both staff who delivered it and men who attended, with participants rating therapeutic alliance and evaluating sessions highly. Despite this, attendance was low. However, by cycle 3 the overall median rate of session attendance had increased to 64.3%. *2) ≥ 60% of eligible male participants recruited.* Seventy-one percent of eligible men were recruited. *3) ≥ 70% of male and female participants followed-up 4-months post-randomisation.* Overall, 49% of men and 63% of women were followed-up. However, by cycle 3 the follow-up rate was 100% for both men and women. *4) Substance use and 5) IPA by men in the intervention group did not increase (average baseline level (with confidence intervals) at 16 weeks post-randomisation follow-up.* Comparisons of baseline and follow-up values showed no worsening in substance use nor IPA in the intervention arm at 16 weeks post-randomisation follow-up (Table S5). Criteria 2, 4 and 5 were met and criteria 1 and 3 were not met, although improvements were demonstrated by the third study cycle. Lessons learned during this feasibility trial will be implemented into the study design and procedures for the definitive trial.

#### Economic evaluation

Total training costs for all three sites were estimated at £36,978, including trainer and facilitator time costs, printing, travel, subsistence, accommodation, and post-training support sessions. Mean delivery cost of pre-group sessions was £35 per participant (*n* = 25) for participants with complete attendance data. The majority of group sessions (58/71) lasted 2 h, the remainder being shorter. Overall, the total group session duration was 126.25 h, and the total cost was £8,585 across six sets of intervention delivery.

Missing service use and quality of life data were mostly due to lost-to-follow-up for both male participants and female partners. Items missing among those followed up was low. Some healthcare services were used more frequently, such as general practitioner service and outpatient appointments. At baseline approximately a fifth of male participants in each arm reported arrest, caution or penalty notices for disorders, or being on probation during the previous 4 months. Fewer than 10 participants in either arm consulted legal services for their cases, most of which were covered by legal aid. Female partners rarely reported contacts with police, criminal justice or legal services. Neither male nor female partners used housing services. Because of the employment status of the population, most participants and partners found questions about absence from work not applicable. Social care was the main cost related to children. Among male participants, the mean costs in the control arm were consistently higher than in the intervention arm. Female participants as compared to male participants tended to report higher costs for children’s healthcare (Table 5).

**Table 5:** Mean costs of substance misuse, healthcare, social services, children’s care, policing and justice system and civil legal services reported by male participants at baseline and 16 weeks follow-up, by arm

	Baseline				16 weeks follow-up			
Costs	Intervention + TAU (***n*** = 54)		TAU only (***n*** = 50)		Intervention + TAU (***n*** = 22)		TAU only (***n*** = 29)	
	**n**	**£, mean (SD)**	**n**	**£, mean (SD)**	**n**	**£, mean (SD)**	**n**	**£, mean (SD)**
**Male participants**								
Substance misuse, healthcare and social services	52	2916 (3108)	47	3267 (4066)	22	1496 (1012)	27	2163 (1283)
Children’s healthcare and social services	54	7 (48)	50	94 (532)	22	70 (284)	28	160 (789)
Children in care	52	2473 (17,830)	49	9769 (64,297)	22	–	29	–
Policing and justice system	54	1581 (2952)	49	2459 (4735)	22	420 (1140)	28	1115 (3411)
Civil legal service	54	126 (419)	50	119 (345)	22	116 (300)	28	91 (355)
**Female current/ex-partners**								
Substance misuse, healthcare and social services	16	1479 (3542)	8	1857 (3275)	11	714 (866)	6	317 (338)
Children’s healthcare and social services	18	279 (879)	9	137 (277)	10	109 (216)	6	9 (22)
Children in care	18	5357 (22,729)	9	–	11	–	6	–
Policing and justice system	18	234 (994)	9	–	11	–	6	–
Civil legal service	18	237 (817)	9	–	11	–	6	–

Male participants scored lowest on the anxiety/depression domain of the EQ-5D-3L. At baseline, only 17% (9/52) in the intervention arm and 14% (7/50) in the control arm reported not feeling anxious or depressed at all. At 16 weeks post-baseline, the proportion not feeling anxious or depressed was still the lowest among five domains: 27% (6/22) in the intervention arm and 17% (5/29) in the control arm. The mean Visual Analogue Scale score from the EQ-5D-3L was 55.2 (sd 23.1) in the intervention arm (*n* = 54) and 55.0 (sd 21.2) in the control arm (*n* = 50) at baseline. At 16 weeks, the mean score was 57.8 (sd 20.1) in the intervention arm (*n* = 22) and 56.0 (sd 21.8) in the control arm (*n* = 29).

The proportion of male participants feeling completely capable on all five capability aspects of ICECAP-A was generally low. Most participants had some level of incapability. The lowest aspect was stability where only one male participant (control arm), at baseline and 16 weeks follow-up respectively, felt settled and secure in all areas of life while the rest did not feel so, at least in some areas of life.

## Discussion

We have demonstrated that it is possible for trained substance use treatment staff to safely deliver the ADVANCE intervention to male IPA perpetrators in substance use treatment services, and for risk to their current or ex-female partners to be effectively managed and mitigated with the use of case management and the support of an integrated support service.

We recruited 104 men to the feasibility trial, the largest sample in a trial with men in substance use treatment to date. A systematic review of interventions to reduce IPA perpetration [23] identified just three (pilot) efficacy/ effectiveness trials [58–60] conducted in substance use treatment services recruiting samples of 52, 63 and 75 respectively.

### Feasibility of conducting a definitive trial

Challenges encountered in the implementation of ADVANCE were consistent with those identified in evaluations of complex interventions notably staffing and contextual issues [61]. Five pre-specified criteria were established to determine whether to progress to an efficacy and cost-effectiveness trial based on at least 60% of eligible male participants being recruited to the trial (met); the intervention being acceptable to staff and male participants (not met); at least 70% of participants followed-up (not met) and no increase in the level of substance use or IPA perpetrated by men in the intervention arm 16-weeks post-randomisation (met). Three of the progression criteria were met and two were not. However, given the improvements in engagement and retention in the intervention and the 100% follow-up rate of both men and women demonstrated by the third study cycle, progression to a definitive trial was supported by the funders. These criteria will be discussed in turn, alongside lessons learned and recommended changes to the study design and procedures in a future efficacy and cost-effectiveness trial.

#### Recruitment

We have shown that men who perpetrate IPA can be recruited and randomized in substance use services, with 71% of eligible men recruited and 100% of consented men randomized. To reduce potential stigma, the study was introduced as providing men with skills to improve their relationships and communication with their partners and reduce disagreements, arguments and abuse. Our eligibility rate of men approached was low (7%), however it is important to note that often the researchers did not get the opportunity to discuss the study in detail with potential participants in waiting rooms. Forty-two percent (42.2%) of the men approached for pre-screening were not interested in hearing about the study or in taking part, and 47.0% inadvertently disclosed they were not eligible in discussion with the researcher about the study or they were deemed unsuitable by their keyworker. Of those who disclosed they were ineligible, 51.9% stated there was no IPA in their relationship or they were not in a relationship; but this was not formally assessed by the researcher. We know from previous research in England that around four in ten men in substance use treatment had perpetrated IPA towards their current partner in the past 12 months [13], therefore we believe that this eligibility rate of the men approached does not reflect need. One European trial of a 16-session integrated intervention delivered in a substance use treatment service used similar recruitment methods to ADVANCE, including a two-stage screening process [58]. They reported a similar eligibility rate to ADVANCE (120 eligible/1799 first stage screened, 7%) but a lower randomisation rate (52 randomised/120 eligible, 43%). We recommend in future trials that eligible men are not approached in waiting rooms by researchers as this proved inefficient, but rather they are identified and screened by substance use treatment staff.

It is not feasible to conduct a future trial of ADVANCE based on partners’ outcomes using the current design. It proved difficult to collect data from female current or ex partners of male participants in the trial, with just 27 partners recruited. While this recruitment rate is low (26%) it is similar to the trial in the Netherlands where 31% of female partners of men attending a group perpetrator intervention in substance use treatment were recruited [58]. Despite being recommended [62], a recent review [23] found that only four trials among perpetrators who use substances had collected outcome data from a female partner, highlighting the difficulty in recruiting and retaining female partners in research on IPA [58–60, 63]. In ADVANCE, 16.3% of men’s partners provided outcome data, compared to 7.7% in the trial of men in treatment for substance use in the Netherlands, who like in ADVANCE were not mandated to treatment [58].

In our trial, partners were first contacted by the integrated support services to offer them support and invite them to hear more about the study from the researchers. Of the attempts by the integrated support service to contact the 104 women, only 62 (59.6%) were contactable and 16 (15.4%) declined to participate potentially due to having ‘moved on’ from the relationship emotionally or believing the research was not relevant to them as they did not consider their partner abusive. Although 46 women told the integrated support worker they were interested in hearing about the study from the researcher, researchers were only able to contact 32 women (69.6%). Of the women contacted by researchers, only 5 (15.6%) declined to participate in the research. Despite the low overall recruitment rate of partners, the inclusion of both current and ex-partners is important as IPA can continue or even increase post-separation [64]. Almost half (49%) of women in the Swedish National Violence Against Women Survey who had experienced IPA during cohabitation had experienced stalking and 10% assault, post-separation from an ex-partner [65]. In our trial, almost a third of male participants (32 men; 30.8%) who reported IPA in the past year stated that their relationship had ended, they were living apart but still had contact with their ex-partner. Our previous research supports this, as men in substance use treatment continued to be involved with their ex-partners for various reasons including that she provided a place to live, their relationship was “on-off” or they maintained contact due to shared children [9]. We therefore defend the need to include ex-partners with whom perpetrators have had recent contact in any future trial, to ensure post-separation IPA is included. Additional contacts’ details should be sought for partners to enable researchers to contact them as refusal rate to participate in the study among those contacted was low. This could include email addresses, contact numbers for friends or relatives or services that she attends. This is a common and necessary method used with research in harder to engage populations [66].

#### Acceptability

While men who attended found the intervention useful and rated the intervention content and therapeutic alliance highly, the median rate of intervention session attendance was 28.6%. The reduction in time from randomisation to individual sessions (46.7 to 17.2 days) and to the first group intervention session (from 46.6 to 39.1 days) across cycles may have impacted on attendance as the median rate of intervention session attendance had increased to 64.3% by cycle 3. The lower attendance rates for cycle 1 may be due to recruitment of men requiring more time than anticipated, potentially resulting in men no longer being contactable, available or interested in participating in the intervention. Improvements in attendance and retention by cycle 3 could also be related to an increase in researcher and staff confidence and comfort with study procedures. We recommend ensuring that the intervention begins shortly after randomisation to retain participants.

#### Engagement

In our trial, 34% of men did not attend any intervention sessions. High drop-out rates (around 40–60%) for perpetrator interventions have been reported, irrespective of the intervention’s format, duration or whether participants used substances [22, 58, 59, 67–69]. We found a greater proportion of men who did not attend any ADVANCE intervention sessions were younger, lived in homeless or temporary accommodation or suffered from depression, anxiety or post-traumatic stress disorder. Similar findings have been reported in other studies, where dropout was associated with younger age; experiencing mental health problems, including substance use; lower income, unemployment or being from a lower socioeconomic or minority ethnic group [22, 58, 67, 70–73].

The proportion of men followed-up 16-weeks post randomisation varied across cycles and by cycle 3, 100% of all participants were followed-up. More assertive follow-up is required to enhance retention in research including establishing an efficient tracing system to locate participants [66, 74].

Some staff believed the intervention was valuable, ‘life changing’ in some instances, although there was a substantial burden on already stretched services. Intervention delivery was relatively cheap compared with training. Since the staff trained could carry on using the skills learnt, the average costs would reduce if the scale of the programme expands.

Attendance and retention in IPA interventions that include motivational strategies are greater than in interventions that do not [18]. Preparation, attendance reminders, and case management have found to be effective engagement strategies in psychotherapy [75]. The following barriers limit engagement in group therapy: not being sufficiently informed, concerns about social interactions and what will happen in the group and negative group dynamics [76]. We recommend the inclusion of individual sessions to motivate participants to change their behaviour and prepare them for what to expect in the group.

While all recruited participants were randomised, one potential reason for drop-out could be that some men were not allocated to receive the intervention. We recommend randomising on a 2:1 basis in future trials to ensure a greater number of men receive the intervention.

#### Follow-up

To improve the follow-up rate, we recommend using more assertive methods of tracking participants: i.e., as well as recording their contact details, contact details and permission to contact family members, friends and services where they are receiving treatment or support [66].

#### Outcomes

We have demonstrated which of our potential outcome measures were understandable and acceptable to participants. Improvements in IPA perpetration were reported in our trial, and there were no worsening of IPA or substance use by men in the intervention arm. Similar findings have been reported in other trials among men in substance use treatment, although no differences were found between the intervention and substance use treatment as usual [23]. Both trials and naturalistic studies report reductions but not eradication of IPA after treatment for substance use alone [12, 23], related to reductions in substance use and improved relationship functioning [12]. A definitive trial is required to test the efficacy of the ADVANCE intervention 12-months post randomisation.

### Lessons learned for future trials

While only three of the five progression criteria were met, we are confident that the improvements in attendance and retention seen by cycle 3 will be sustained in a future trial of the intervention due to the increased staff capabilities and researcher confidence with study procedures. Lessons learned from the current study will be implemented to increase attendance and engagement in the intervention. We will select services where facilitators and staff were more confident in managing risk and had experience delivering group work to similar populations. We will also ensure a whole service buy-in facilitated by having a service champion. Training on IPA as well as on ADVANCE will now be provided to all substance use treatment staff to improve buy-in. The intervention has been refined based on feedback from staff and participants. The revised intervention will include a group introductory session to prepare men for group, in an attempt to increase engagement and retention. Substance use treatment staff will be responsible for screening men which should enhance engagement and reduce the time from randomisation to the intervention starting, which we have demonstrated increases intervention attendance.

## Conclusions

It was feasible to recruit and follow-up men in substance use treatment who perpetrate IPA to a trial of a perpetrator intervention and to manage the risk to their female current or ex-partners throughout the intervention. Although intervention attendance was low, strategies have been implemented to improve recruitment, engagement and retention in an efficacy trial. It was not feasible to conduct a future trial of ADVANCE based on current or ex-female partners’ outcomes using the current design.

Due to Covid-19 restrictions, the ADVANCE intervention is currently being iteratively adapted for technology enabled delivery. The feasibility and acceptability of delivering a technology enabled version of ADVANCE will be assessed.

## Supplementary Information

**Additional file 1: Table S1.** Description of participant-centred outcome measures and their completeness, acceptability and understanding ratings at baseline. **Table S2.** Baseline measures of the female participants in the ADVANCE feasibility trial (*n* = 27). **Table S3.** Outcome measures of the female participants in the ADVANCE feasibility trial (*n* = 27). **Table S4.** Estimated treatment differences for male participants at 16 weeks follow-up. **Table S5.** Process variables to describe progression criteria.

## Data Availability

All data generated or analysed during this study are included in this published article and its supplementary information files.

## Abbreviations

95%CI: 95% confidence interval
ABI-R: Revised Abusive Behavior Inventory
ACE: Adverse Childhood Experience
AUDIT: Alcohol Use Disorders Identification Test
BIDR-16: Balanced Inventory of Desirable Responding Short Form
BSC: Brief Self Control Scale
CALPAS-P: California Psychotherapy Alliance Scale-Short Form
CBS-R: Revised Controlling Behaviours Scale
CONSORT: Consolidated Standards of Reporting Trials
CPQ-SF: Communications Patterns Questionnaire-Short Form
DUDIT: Drug Use Disorders Identification Test
EQ-5D-3L: European Quality of life 5 Dimensions-3 Level
GAD-7: General Anxiety Disorder-7
ICECAP-A: ICEpop CAPability measure for Adults
IPA: Intimate partner abuse
IPVRAS: Partner Violence Responsibility Attribution Scale
PAS: Propensity for Abusiveness Scale
PC-PTSD-5: Primary Care PTSD Screen for DSM-5
PHQ-9: Patient Health Questionnaire-9
SAPAS: Standardised Assessment of Personality—Abbreviated Scale
TAU: treatment as usual
URICA-DV: University of Rhode Island Change Assessment for Domestic Violence Offenders-Revised
WAI-SR: Working Alliance Inventory – Short Revised
