# Supplementary material for: ADVANCE integrated group intervention to address both substance use and intimate partner abuse perpetration by men in substance use treatment: a feasibility randomised controlled trial
Source: BMC Public Health. 2021 May 25;21:980. doi: 10.1186/s12889-021-11012-3 (PMC8147906; doi:10.1186/s12889-021-11012-3)
Supplement: Supplementary file 1 — Additional file 1: Table S1. Description of participant-centred outcome measures and their completeness, acceptability and understanding ratings at baseline. Table S2. Baseline measures of the female participants in the ADVANCE feasibility trial (n = 27). Table S3. Outcome measures of the female participants in the ADVANCE feasibility trial (n = 27). Table S4. Estimated treatment differences for male participants at 16 weeks follow-up. Table S5. Process variables to describe progression criteria. [file 12889_2021_11012_MOESM1_ESM.docx]

### **Table S1.** **Description of participant-centred outcome measures and their completeness, acceptability and understanding ratings at baseline**

|  | **Instrument** | **Content** | **Scoring** | **Completeness**  **N (%)** | | **Acceptability rating**  **median (LQ- UQ)** | | **Understandability rating**  **median (LQ- UQ)** | |
| --- | --- | --- | --- | --- | --- | --- | --- | --- | --- |
|  |  |  |  | **Male participants**  **(n=104)** | **Female (ex) partners**  **(n=27)** | **Male participants**  **(n=104)** | **Female (ex) partners**  **(n=27)** | **Male participants**  **(n=104)** | **Female (ex) partners**  **(n=27)** |
| **Substance use** | Treatment Outcome Profile (44) | Number of days substances used in each of the past 4 weeks | Mean number of days (each) substance is used in each of the past 4 weeks | 104 (100.0) | 26 (96.3) | 3.0 (3.0-3.0) | 3.0 (3.0-3.0) | 3.0 (3.0-3.0) | 3.0 (3.0-3.0) |
|  | The Addiction Severity Index (45) | Number of days in the past 4 weeks that problems with particular substances were experienced | Mean number of days in the past 4 weeks that problems with particular substances were experienced | 104 (100.0) | 26 (96.3) | 3.0 (3.0-3.0) | 3.0 (3.0-3.0) | 3.0 (3.0-3.0) | 3.0 (3.0-3.0) |
| **Mental heath** | Patient Health Questionnaire (PHQ-9) (46) | 9-item measuring depressive  symptoms  in past 2 weeks | Score range 0-27. Score ≥ 10 cut off for major depression. | 104 (100.0) | 27 (100.0) | 3.0 (3.0-3.0) | 3.0 (3.0-3.0) | 3.0 (3.0-3.0) | 3.0 (3.0-3.0) |
|  | The Generalised Anxiety Disorder Assessment  (GAD-7)  (47) | 7-item measuring general anxiety symptoms in past 2 weeks | Score range 0-21. Score of ≥ 10 cut off for GAD case | 104 (100.0) | 27 (100.0) | 3.0 (3.0-3.0) | 3.0 (3.0-3.0) | 3.0 (3.0-3.0) | 3.0 (3.0-3.0) |
|  | The Primary Care Post-Traumatic Stress Disorder Screen (PC-PTSD-5) (48) | 5-item screen for PTSD in past month | Score range 0-5. Score of ≥ 3 indicates PTSD | 102 (98.1) | 27 (100.0) | 3.0 (3.0-3.0) | 3.0 (3.0-3.0) | 3.0 (3.0-3.0) | 3.0 (3.0-3.0) |
| **Intimate partner abuse** | The Abusive Behaviour Inventory Revised (ABI-R) [perpetration] (30) | 25-item reliable tool to measure perpetration of physical (13 items), psychological (9 items), and sexual abuse (3 items) | Each item can be scored from 1 (never) to 5 (very frequently). The higher the score on each subscale, the greater the frequency of abuse perpetration. | 104 (100.0) | 27 (100.0) | 3.0 (2.0-3.0) | 3.0 (3.0-3.0) | 3.0 (3.0-3.0) | 3.0 (3.0-3.0) |
|  | The Abusive Behaviour Inventory Revised (ABI-R) [victimisation] (30) | 25-item reliable tool to measure experience of physical (13 items), psychological (9 items), and sexual abuse (3 items) | Each item can be scored from 1 (never) to 5 (very frequently). The higher the score on each subscale, the greater the frequency of abuse victimisation. | 103 (99.0) | 27 (100.0) | 3.0 (2.0-3.0) | 3.0 (3.0-3.0) | 3.0 (3.0-3.0) | 3.0 (3.0-3.0) |
|  | Revised Controlling Behaviours Scale (CBS-R) [perpetration] (32) | Four adapted questions from the 24 item CBS-R: smash your partner’s property when annoyed/angry; want to know where your partner went and who they spoke to when not together; tell your partner they were going mad; and try to restrict time your partner spent with family or friends | Total score ranges from 0 to 16; the higher the score, the greater the frequency of perpetrating controlling behaviours | 104 (100.0) | 27 (100.0) | 3.0 (2.0-3.0) | 3.0 (3.0-3.0) | 3.0 (3.0-3.0) | 3.0 (3.0-3.0) |
|  | Revised Controlling Behaviours Scale (CBS-R) [victimisation] (32) | Four adapted questions from the 24 item CBS-R: partner smashed your property when annoyed/angry; partner wanted to know where you went and who you spoke to when not together; partner told you that you were going mad; and partner tried to restrict time you spent with family or friends | Total score ranges from 0 to 16; the higher the score, the greater the frequency of a partner using controlling behaviours | 103 (99.0) | 27 (100.0) | 3.0 (3.0-3.0) | 3.0 (3.0-3.0) | 3.0 (3.0-3.0) | 3.0 (3.0-3.0) |
|  | The Communications Patterns Questionnaire-Short Form (CPQ-SF) (36) | 11-item self-assessment of spouses’ perceptions of relationship conflict and communication patterns. The CPQ-SF has 4 subscales: wife demand/ husband withdraw; husband demand/ wife withdraw; total demand/ withdraw; and overall positive interaction | Higher scores on each subscale suggest which communication pattern is likely to be used during conflict interactions. | 101 (97.1) | 26 (96.3) | 3.0 (2.0-3.0) | 3.0 (3.0-3.0) | 3.0 (2.0-3.0) | 3.0 (3.0-3.0) |
|  | Intimate Partner Violence Responsibility Attribution Scale (IPVRAS) (37)* | Two scales (9 items) assessed perpetrators’ responsibility attribution to the victim (V) and responsibility attribution to the offender's personal context (O) | The higher the score, the more responsibility attributed to the victim (V) or offender’s personal context (O). | 86 (82.7) | n/a | 2.0 (1.0-3.0) | n/a | 3.0 (3.0-3.0) | n/a |
|  | Propensity for Abusiveness Scale (PAS) (38)* | 12-item anger subscale | Scores range from 12 (completely undescriptive of you) to 60 (completely descriptive of you) | 103 (99.0) | n/a | 3.0 (3.0-3.0) | n/a | 3.0 (3.0-3.0) | n/a |
|  | Other perpetration | 4 questions on using children against a partner (32); 3 questions on the use of technology to perpetrate abuse (33); 1 on stalking (34); 1 on being locked in against will (34) | The higher the score, the greater the frequency of perpetrating the behaviour towards a partner | 104 (100.0) | 27 (100.0) | 3.0 (2.0-3.0) | 3.0 (3.0-3.0) | 3.0 (3.0-3.0) | 3.0 (3.0-3.0) |
|  | Other victimisation | 4 questions on partner using children against them (32); 3 questions on partner using technology to abuse (33); 1 on stalking (34); 1 on being locked in against will (34) | The higher the score, the greater the frequency of experiencing the behaviour from a partner | 100 (96.2) | 27 (100.0) | 3.0 (2.5-3.0) | 3.0 (3.0-3.0) | 3.0 (3.0-3.0) | 3.0 (3.0-3.0) |
| **Self-management** | Brief Self-Control Scale  (BSCS) (39)* | 13-item scale enquires about typical dispositional self-regulatory behaviours using two factors of self-control: restraint and impulsivity | Total scores range from 13 (not at all like me) to 65 (very much like me) | 104 (100.0) | n/a | 3.0 (3.0-3.0) | n/a | 3.0 (3.0-3.0) | n/a |
| **Desirable responding** | The Balanced Inventory of Desirable Responding Short Form (BIDR-16) (40)* | 16 item scale that consists of two subscales: Self-  Deceptive Enhancement (honest but overly positive  responding) and Impression Management (bias toward  pleasing others or ‘deliberate self-presentation’). | Total scores range from 16 to 128. Higher  scores indicating more desirable responses. | 104 (100.0) | n/a | 3.0 (2.5-3.0) | n/a | 3.0 (3.0-3.0) | n/a |
| **Motivation to change behaviour** | The URICA-Domestic Violence (URICA-DV) (41)* | 32-item scale measuring  perpetrators’ readiness to end their violence.  URICA-DV includes four subscales to measure stages of  change pre-contemplation, contemplation, action, and  relapse. | Responses are given on a 5-point Likert scale  ranging from 1 (strong disagreement) to 5 (strong  agreement). | 83 (79.8) | n/a | 1.0 (1.0-3.0) | n/a | 3.0 (2.0-3.0) | n/a |
| **Quality of Life** | ICEpop CAPability measure for Adults (ICECAP-A) (57) | Assessed 5 attributes of well-being using a capability approach:  attachment; stability; achievement; enjoyment and autonomy | Tariff values range from 1 (full capability) to 0 (no capability). | 102 (98.1) | 27 (100.0) | 3.0 (3.0-3.0) | 3.0 (3.0-3.0) | 3.0 (3.0-3.0) | 3.0 (3.0-3.0) |
|  | EQ-5D-3L (56) | A descriptive system assesses 5 dimensions of health state (mobility, self-care, usual activities, pain/discomfort, and anxiety/depression) on the day of administering. The Visual Analogue Scale (VAS) records current self-rated health on a vertical visual analogue scale | The tariff index score ranges from 1 (perfect health) to – 0.594 (worst health), with death anchored at 0. The VAS current health state is rated from 0 (worst imaginable) to 100 (best imaginable health) | 101 (97.1) | 27 (100.0) | 3.0 (3.0-3.0) | 3.0 (3.0-3.0) | 3.0 (3.0-3.0) | 3.0 (3.0-3.0) |
| **Healthcare, social, and other legal and civil services use and criminal justice contacts** | Self-report questionnaire | Utilisation of primary and secondary healthcare, social services, and other legal and civil services, including the prescribing of medication, and their contacts with criminal justice were recorded | Service utilisation and prescriptions transformed to costs using local unit costs | 96 (92.3) | 24 (88.9) | 3.0 (3.0-3.0) | 3.0 (3.0-3.0) | 3.0 (3.0-3.0) | 3.0 (3.0-3.0) |
| *LQ= lower quartile, UQ = upper quartile,*  **Collected on male participants only* | | | | | | | | | |

### **Table S2: Baseline measures of the female participants in the ADVANCE feasibility trial (n=27)**

| **Variable** |  | **Trial Arm** | | **Total**  **(n=27)** |
| --- | --- | --- | --- | --- |
|  |  | **Intervention + TAU**  **(n=18)** | **TAU only**  **(n=9)** |  |
| Age at consent date (years) - mean (sd) (N = 27) |  | 39.3 (11.8) | 46.8 (11.8) | 41.8 (12.1) |
| Ethnic group - n (%) (N = 27) | White | 10 (55.6) | 6 (66.7) | 16 (59.3) |
|  | Black | 3 (16.7) | 0 (0.0) | 3 (11.1) |
|  | Asian | 3 (16.7) | 2 (22.2) | 5 (18.5) |
|  | Other | 2 (11.1) | 1 (11.1) | 3 (11.1) |
| Highest qualification level - n (%) (N = 27) | 1. No formal qualifications | 2 (11.1) | 2 (22.2) | 4 (14.8) |
|  | 2. O-levels, CSEs, GCSEs, O grades, Standard grades | 6 (33.3) | 2 (22.2) | 8 (29.6) |
|  | 3. NVQ Levels 1-3/GNVQ | 4 (22.2) | 1 (11.1) | 5 (18.5) |
|  | 4. A levels, AS levels, Higher School Certificate | 2 (11.1) | 0 (0.0) | 2 (7.4) |
|  | 5. NVQ levels 4-5, HNC, HND | 2 (11.1) | 0 (0.0) | 2 (7.4) |
|  | 6. Degree or higher degree | 2 (11.1) | 3 (33.3) | 5 (18.5) |
|  | 7. Other qualifications (e.g. City and Guilds, RSA/OCR, BTEC/Edexcel) | 0 (0.0) | 1 (11.1) | 1 (3.7) |
| Employment status - n (%) (N = 27) | 1. Employed | 4 (22.2) | 5 (55.6) | 9 (33.3) |
|  | 2. Looking after your home/family | 5 (27.8) | 0 (0.0) | 5 (18.5) |
|  | 3. Unemployed and looking for work (Jobseekers' Allowance) | 2 (11.1) | 0 (0.0) | 2 (7.4) |
|  | 4. Unable to work due to long term sickness (Employment and Support Allowance... | 5 (27.8) | 1 (11.1) | 6 (22.2) |
|  | 5. Retired from paid work | 1 (5.6) | 1 (11.1) | 2 (7.4) |
|  | 6. In full time education | 0 (0.0) | 0 (0.0) | 0 (0.0) |
|  | 7. Other | 1 (5.6) | 2 (22.2) | 3 (11.1) |
| Living arrangements - n (%) (N = 27) | 1. Sleeping rough on streets/parks | 0 (0.0) | 0 (0.0) | 0 (0.0) |
|  | 2. In a hostel or supported accommodation | 3 (16.7) | 0 (0.0) | 3 (11.1) |
|  | 3. Squatting | 0 (0.0) | 0 (0.0) | 0 (0.0) |
|  | 4. Sleeping on somebody's sofa/floor | 0 (0.0) | 0 (0.0) | 0 (0.0) |
|  | 5. In emergency accommodation, e.g. nightshelter, refuge | 0 (0.0) | 0 (0.0) | 0 (0.0) |
|  | 6. In B&B or other temporary accommodation | 1 (5.6) | 0 (0.0) | 1 (3.7) |
|  | 7. Housed - in own tenancy | 10 (55.6) | 4 (44.4) | 14 (51.9) |
|  | 8. Housed - in someone else's tenancy | 2 (11.1) | 2 (22.2) | 4 (14.8) |
|  | 9. Other | 2 (11.1) | 3 (33.3) | 5 (18.5) |
| Sexuality - n (%) (N = 27) | 1. Heterosexual or straight | 18 (100.0) | 9 (100.0) | 27 (100.0) |
|  | 2. Gay | 0 (0.0) | 0 (0.0) | 0 (0.0) |
|  | 3. Bisexual | 0 (0.0) | 0 (0.0) | 0 (0.0) |
|  | 4. Other | 0 (0.0) | 0 (0.0) | 0 (0.0) |
|  | 5. Do not know | 0 (0.0) | 0 (0.0) | 0 (0.0) |
|  | 6. Prefer not to say | 0 (0.0) | 0 (0.0) | 0 (0.0) |
| Relationship status with current/ex partner now - n (%) (N = 27) | 1. Together and living together | 10 (55.6) | 5 (55.6) | 15 (55.6) |
|  | 2. Together but living apart | 1 (5.6) | 1 (11.1) | 2 (7.4) |
|  | 3. In the process of splitting up | 1 (5.6) | 1 (11.1) | 2 (7.4) |
|  | 4. The relationship has ended and we are living apart with no contact | 1 (5.6) | 0 (0.0) | 1 (3.7) |
|  | 5. The relationship has ended and we are living apart and still have contact | 3 (16.7) | 0 (0.0) | 3 (11.1) |
|  | 6. I am not sure | 0 (0.0) | 0 (0.0) | 0 (0.0) |
|  | 7. Something else | 2 (11.1) | 2 (22.2) | 4 (14.8) |
| What are your hopes for your relationship with them in the future? - n (%) (N = 27) | 1. That we will be together and living together | 10 (55.6) | 5 (55.6) | 15 (55.6) |
|  | 2. That this relationship will end | 1 (5.6) | 0 (0.0) | 1 (3.7) |
|  | 3. I am not sure | 1 (5.6) | 2 (22.2) | 3 (11.1) |
|  | 4. I am in another relationship already | 0 (0.0) | 0 (0.0) | 0 (0.0) |
|  | 5. Something else | 6 (33.3) | 2 (22.2) | 8 (29.6) |
| Any children? - n (%) (N = 27) | No | 6 (33.3) | 5 (55.6) | 11 (40.7) |
|  | Yes | 12 (66.7) | 4 (44.4) | 16 (59.3) |
| Face to face contact with current/ex partner in past 4 months - n (%) (N = 27) | No | 0 (0.0) | 0 (0.0) | 0 (0.0) |
|  | Yes | 18 (100.0) | 9 (100.0) | 27 (100.0) |
| Telephone contact (speaking) with current/ex partner in past 4 months - n (%) (N = 27) | No | 0 (0.0) | 1 (11.1) | 1 (3.7) |
|  | Yes | 18 (100.0) | 8 (88.9) | 26 (96.3) |
| Telephone contact (text) with current/ex partner in past 4 months - n (%) (N = 27) | No | 2 (11.1) | 1 (11.1) | 3 (11.1) |
|  | Yes | 16 (88.9) | 8 (88.9) | 24 (88.9) |
| Social media contact with current/ex partner in past 4 months - n (%) (N = 27) | No | 13 (72.2) | 8 (88.9) | 21 (77.8) |
|  | Yes | 5 (27.8) | 1 (11.1) | 6 (22.2) |
| AUDIT Total Score - mean (sd) (N = 27) |  | 5.3 (7.1) | 5.7 (10.7) | 5.4 (8.3) |
| Hazardous and harmful alcohol use (AUDIT scale 8 points or more) - n (%) (N = 27) | No | 12 (66.7) | 8 (88.9) | 20 (74.1) |
|  | Yes | 6 (33.3) | 1 (11.1) | 7 (25.9) |
| DUDIT Total Score - mean (sd) (N = 26) |  | 8.3 (13.6) | 6.3 (11.6) | 7.7 (12.8) |
| Highly probable dependent on one or more drugs (DUDIT scale 25 points or more) - n (%) (N = 26) | No | 15 (83.3) | 7 (87.5) | 22 (84.6) |
|  | Yes | 3 (16.7) | 1 (12.5) | 4 (15.4) |
| Treatment for Heroin - n (%) (N = 27) | No | 15 (83.3) | 7 (77.8) | 22 (81.5) |
|  | Yes | 3 (16.7) | 2 (22.2) | 5 (18.5) |
| Treatment for Cocaine - n (%) (N = 27) | No | 18 (100.0) | 9 (100.0) | 27 (100.0) |
|  | Yes | 0 (0.0) | 0 (0.0) | 0 (0.0) |
| Treatment for Crack - n (%) (N = 27) | No | 16 (88.9) | 8 (88.9) | 24 (88.9) |
|  | Yes | 2 (11.1) | 1 (11.1) | 3 (11.1) |
| Treatment for Cannabis - n (%) (N = 27) | No | 18 (100.0) | 9 (100.0) | 27 (100.0) |
|  | Yes | 0 (0.0) | 0 (0.0) | 0 (0.0) |
| Treatment for Alcohol - n (%) (N = 27) | No | 16 (88.9) | 9 (100.0) | 25 (92.6) |
|  | Yes | 2 (11.1) | 0 (0.0) | 2 (7.4) |
| Treatment episode length - n (%) (N = 7) | 1. Less than 6 months | 1 (20.0) | 0 (0.0) | 1 (14.3) |
|  | 2. 6-12 months | 0 (0.0) | 1 (50.0) | 1 (14.3) |
|  | 3. More than 12 months | 4 (80.0) | 1 (50.0) | 5 (71.4) |
| SAPAS Total score - mean (sd) (N = 27) |  | 3.7 (1.7) | 2.8 (1.4) | 3.4 (1.7) |
| SAPAS Score of 3 or more - n (%) (N = 27) | No | 4 (22.2) | 3 (33.3) | 7 (25.9) |
|  | Yes | 14 (77.8) | 6 (66.7) | 20 (74.1) |
| Adverse Childhood Experiences (ACE) Total Score - mean (sd) (N = 26) |  | 3.8 (2.7) | 2.0 (2.3) | 3.3 (2.7) |

## Table S3: Outcome measures of the female participants in the ADVANCE feasibility trial (n=27)

| **Variable** | |  | **Baseline** | | | **16 weeks follow-up** | | |
| --- | --- | --- | --- | --- | --- | --- | --- | --- |
|  |  |  | **Intervention + TAU**  **(n=18)** | **TAU only**  **(n=9)** | **Total**  **(n=27)** | **Intervention + TAU**  **(n=11)** | **TAU only**  **(n=6)** | **Total**  **(n=17)** |
| **Substance Use** | No. of days of alcohol use in the past 28 days  *Scale Range 0-28* | N | 18 | 9 | 27 | 11 | 6 | 17 |
|  |  | median (LQ-UQ) | 0.0 (0.0-6.0) | 1.0 (0.0-18.0) | 0.0 (0.0-8.0) | 0.0 (0.0-4.0) | 2.0 (0.0-24.0) | 0.0 (0.0-4.0) |
|  | No. of days of heroin use in the past 28 days  *Scale Range 0-28* | N | 17 | 9 | 26 | 11 | 6 | 17 |
|  |  | median (LQ-UQ) | 0.0 (0.0-0.0) | 0.0 (0.0-0.0) | 0.0 (0.0-0.0) | 0.0 (0.0-0.0) | 0.0 (0.0-0.0) | 0.0 (0.0-0.0) |
|  | No. of days of crack use in the past 28 days  *Scale Range 0-28* | N | 17 | 9 | 26 | 11 | 6 | 17 |
|  |  | median (LQ-UQ) | 0.0 (0.0-0.0) | 0.0 (0.0-0.0) | 0.0 (0.0-0.0) | 0.0 (0.0-0.0) | 0.0 (0.0-0.0) | 0.0 (0.0-0.0) |
|  | No. of days problems experienced with alcohol  *Scale Range 0-28* | N | 18 | 9 | 27 | 11 | 6 | 17 |
|  |  | median (LQ-UQ) | 0.0 (0.0-0.0) | 0.0 (0.0-0.0) | 0.0 (0.0-0.0) | 0.0 (0.0-0.0) | 0.0 (0.0-0.0) | 0.0 (0.0-0.0) |
|  | No. of days problems experienced with heroin  *Scale Range 0-28* | N | 17 | 9 | 26 | 11 | 6 | 17 |
|  |  | median (LQ-UQ) | 0.0 (0.0-0.0) | 0.0 (0.0-0.0) | 0.0 (0.0-0.0) | 0.0 (0.0-0.0) | 0.0 (0.0-0.0) | 0.0 (0.0-0.0) |
|  | No. of days problems experienced with crack  *Scale Range 0-28* | N | 17 | 9 | 26 | 11 | 6 | 17 |
|  |  | median (LQ-UQ) | 0.0 (0.0-0.0) | 0.0 (0.0-0.0) | 0.0 (0.0-0.0) | 0.0 (0.0-0.0) | 0.0 (0.0-0.0) | 0.0 (0.0-0.0) |
| **Mental Health** | PHQ-9 Total Score  *Scale Range 0-27* | N | 18 | 9 | 27 | 11 | 6 | 17 |
|  |  | mean (sd) | 10.8 (7.6) | 9.3 (4.8) | 10.3 (6.7) | 7.0 (6.4) | 4.5 (3.7) | 6.1 (5.6) |
|  | PHQ-9 score 10 or more | n (%) | 9 (50.0) | 6 (66.7) | 15 (55.6) | 3 (27.3) | 1 (16.7) | 4 (23.5) |
|  | GAD-7 Total Score  *Scale Range 0-21* | N | 18 | 9 | 27 | 11 | 6 | 17 |
|  |  | mean (sd) | 10.4 (6.8) | 7.1 (4.5) | 9.3 (6.3) | 5.7 (6.6) | 3.3 (2.7) | 4.9 (5.6) |
|  | GAD-7 score 10 or more | n (%) | 9 (50.0) | 3 (33.3) | 12 (44.4) | 2 (18.2) | 0 (0.0) | 2 (11.8) |
|  | Primary Care PTSD Screen Total Score  *Scale Range 0-5* | N | 18 | 9 | 27 | 11 | 6 | 17 |
|  |  | median (LQ-UQ) | 2.0 (0.0-6.0) | 0.0 (0.0-2.0) | 2.0 (0.0-4.0) | 1.0 (0.0-3.0) | 0.0 (0.0-1.0) | 1.0 (0.0-2.0) |
|  | PTSD Screen score 3 or more | n (%) | 8 (44.4) | 2 (22.2) | 10 (37.0) | 3 (27.3) | 1 (16.7) | 4 (23.5) |
| **Intimate Partner Abuse in the past 4 months** | ABI-R Perpetration Total Score  *Scale Range 25-125* | N | 18 | 9 | 27 | 11 | 6 | 17 |
|  |  | median (LQ-UQ) | 34.5 (29.0-41.0) | 35.0 (29.0-40.0) | 35.0 (29.0-41.0) | 29.0 (27.0-33.0) | 29.5 (28.0-35.0) | 29.0 (27.0-33.0) |
|  | ABI-R Perpetration Physical Score  *Scale Range 9-45* | N | 18 | 9 | 27 | 11 | 6 | 17 |
|  |  | median (LQ-UQ) | 9.0 (9.0-11.0) | 10.0 (9.0-11.0) | 9.0 (9.0-11.0) | 9.0 (9.0-9.0) | 9.0 (9.0-9.0) | 9.0 (9.0-9.0) |
|  | ABI-R Perpetration Psychological Score  *Scale Range 13-65* | N | 18 | 9 | 27 | 11 | 6 | 17 |
|  |  | median (LQ-UQ) | 22.0 (17.0-27.0) | 21.0 (17.0-26.0) | 22.0 (17.0-27.0) | 17.0 (15.0-21.0) | 17.0 (16.0-23.0) | 17.0 (15.0-21.0) |
|  | ABI-R Perpetration Sexual Score  *Scale Range 3-15* | N | 18 | 9 | 27 | 11 | 6 | 17 |
|  |  | median (LQ-UQ) | 3.0 (3.0-3.0) | 3.0 (3.0-3.0) | 3.0 (3.0-3.0) | 3.0 (3.0-3.0) | 3.0 (3.0-3.0) | 3.0 (3.0-3.0) |
|  | ABI-R Victimisation Total Score  *Scale Range 25-125* | N | 18 | 9 | 27 | 11 | 6 | 17 |
|  |  | median (LQ-UQ) | 38.0 (32.0-51.0) | 38.0 (36.0-40.0) | 38.0 (32.0-51.0) | 30.0 (27.0-40.0) | 32.0 (27.0-36.0) | 31.0 (27.0-36.0) |
|  | ABI-R Victimisation Physical Score  *Scale Range 9-45* | N | 18 | 9 | 27 | 11 | 6 | 17 |
|  |  | median (LQ-UQ) | 11.5 (9.0-15.0) | 9.0 (9.0-13.0) | 9.0 (9.0-14.0) | 9.0 (9.0-11.0) | 9.5 (9.0-12.0) | 9.0 (9.0-11.0) |
|  | ABI-R Victimisation Psychological Score  *Scale Range 13-65* | N | 18 | 9 | 27 | 11 | 6 | 17 |
|  |  | median (LQ-UQ) | 24.5 (20.0-34.0) | 23.0 (22.0-28.0) | 24.0 (20.0-33.0) | 17.0 (15.0-25.0) | 19.5 (15.0-21.0) | 17.0 (15.0-21.0) |
|  | ABI-R Victimisation Sexual Score  *Scale Range 3-15* | N | 18 | 9 | 27 | 11 | 6 | 17 |
|  |  | median (LQ-UQ) | 3.0 (3.0-3.0) | 3.0 (3.0-4.0) | 3.0 (3.0-3.0) | 3.0 (3.0-3.0) | 3.0 (3.0-3.0) | 3.0 (3.0-3.0) |
|  | Controlling Behaviours Scale (Partial) Perpetration Total Score  Scale Range 0-16 | N | 18 | 9 | 27 | 11 | 6 | 17 |
|  |  | median (LQ-UQ) | 4.0 (1.0-7.0) | 2.0 (2.0-4.0) | 3.0 (1.0-6.0) | 0.0 (0.0-3.0) | 1.5 (0.0-4.0) | 0.0 (0.0-3.0) |
|  | Controlling Behaviours Scale (Partial) Victimisation Total Score  *Scale Range 0-16* | N | 18 | 9 | 27 | 11 | 6 | 17 |
|  |  | median (LQ-UQ) | 1.5 (0.0-4.0) | 0.0 (0.0-2.0) | 1.0 (0.0-4.0) | 0.0 (0.0-6.0) | 0.5 (0.0-2.0) | 0.0 (0.0-5.0) |
|  | CPQ-SF Male demand/female withdraw total score  *Scale Range 3-27* | N | 18 | 8 | 26 | 11 | 5 | 16 |
|  |  | mean (sd) | 12.6 (6.6) | 8.6 (6.1) | 11.4 (6.6) | 10.5 (6.8) | 8.4 (3.6) | 9.9 (6.0) |
|  | CPQ-SF Female demand/male withdraw total score  *Scale Range 3-27* | N | 18 | 8 | 26 | 11 | 5 | 16 |
|  |  | mean (sd) | 15.6 (5.9) | 13.4 (6.4) | 14.9 (6.0) | 10.7 (6.8) | 12.6 (5.6) | 11.3 (6.3) |
|  | CPQ-SF original demand/withdraw total score  *Scale Range 6-54* | N | 18 | 8 | 26 | 11 | 5 | 16 |
|  |  | mean (sd) | 28.2 (11.1) | 22.0 (8.8) | 26.3 (10.7) | 21.3 (10.6) | 21.0 (8.7) | 21.2 (9.8) |
|  | CPQ-SF alternate demand/withdraw total score  *Scale Range 5-45* | N | 18 | 8 | 26 | 11 | 5 | 16 |
|  |  | mean (sd) | 22.7 (7.4) | 20.6 (5.4) | 22.0 (6.8) | 17.6 (7.5) | 17.0 (6.7) | 17.4 (7.1) |
|  | CPQ-SF criticize/defend total score  *Scale Range 3-27* | N | 18 | 8 | 26 | 11 | 5 | 16 |
|  |  | mean (sd) | 15.8 (8.0) | 10.9 (5.0) | 14.3 (7.5) | 11.8 (6.7) | 10.8 (7.6) | 11.5 (6.7) |
|  | CPQ-SF positive interaction total score  *Scale Range 3-27* | N | 18 | 8 | 26 | 11 | 5 | 16 |
|  |  | mean (sd) | 18.2 (6.0) | 16.6 (6.4) | 17.7 (6.1) | 15.7 (7.7) | 20.8 (4.4) | 17.3 (7.1) |
|  | Use of social media perpetration total score  *Scale Range 2-10* | N | 18 | 9 | 27 | 11 | 6 | 17 |
|  |  | median (LQ-UQ) | 2.0 (2.0-2.0) | 2.0 (2.0-2.0) | 2.0 (2.0-2.0) | 2.0 (2.0-2.0) | 2.0 (2.0-2.0) | 2.0 (2.0-2.0) |
|  | Using children against partner perpetration total score  *Scale Range 4-20* | N | 18 | 9 | 27 | 11 | 6 | 17 |
|  |  | median (LQ-UQ) | 4.0 (4.0-4.0) | 4.0 (4.0-6.0) | 4.0 (4.0-4.0) | 4.0 (4.0-4.0) | 4.0 (4.0-4.0) | 4.0 (4.0-4.0) |
|  | Locked in perpetration total score  *Scale Range 1-5* | N | 18 | 9 | 27 | 11 | 6 | 17 |
|  |  | median (LQ-UQ) | 1.0 (1.0-1.0) | 1.0 (1.0-1.0) | 1.0 (1.0-1.0) | 1.0 (1.0-1.0) | 1.0 (1.0-1.0) | 1.0 (1.0-1.0) |
|  | Stalking perpetration total score  *Scale Range 2-10* | N | 18 | 9 | 27 | 11 | 6 | 17 |
|  |  | median (LQ-UQ) | 2.0 (2.0-4.0) | 2.0 (2.0-3.0) | 2.0 (2.0-4.0) | 2.0 (2.0-4.0) | 2.0 (2.0-2.0) | 2.0 (2.0-2.0) |
|  | Use of social media victimisation total score  *Scale Range 2-10* | N | 18 | 9 | 27 | 11 | 6 | 17 |
|  |  | median (LQ-UQ) | 2.0 (2.0-4.0) | 3.0 (2.0-3.0) | 2.0 (2.0-4.0) | 2.0 (2.0-3.0) | 2.0 (2.0-3.0) | 2.0 (2.0-3.0) |
|  | Using children against partner victimisation total score  *Scale Range 4-20* | N | 18 | 9 | 27 | 9 | 6 | 15 |
|  |  | median (LQ-UQ) | 4.0 (4.0-4.0) | 4.0 (4.0-6.0) | 4.0 (4.0-5.0) | 4.0 (4.0-4.0) | 5.5 (4.0-8.0) | 4.0 (4.0-7.0) |
|  | Locked in victimisation total score  *Scale Range 1-5* | N | 18 | 9 | 27 | 11 | 6 | 17 |
|  |  | median (LQ-UQ) | 1.0 (1.0-1.0) | 1.0 (1.0-1.0) | 1.0 (1.0-1.0) | 1.0 (1.0-1.0) | 1.0 (1.0-1.0) | 1.0 (1.0-1.0) |
|  | Stalking victimisation total score  *Scale Range 2-10* | N | 18 | 9 | 27 | 11 | 6 | 17 |
|  |  | median (LQ-UQ) | 2.0 (2.0-4.0) | 3.0 (2.0-5.0) | 2.0 (2.0-5.0) | 2.0 (2.0-3.0) | 2.0 (2.0-6.0) | 2.0 (2.0-3.0) |

**Table S4: Estimated treatment differences for male participants at 16 weeks follow-up**

| **Outcome measure** | **n** | **Estimated difference (intervention-control)** | **95% Confidence interval** |
| --- | --- | --- | --- |
| PHQ-9 Total Score | 51 | -0.49 | -3.81 to 2.83 |
| GAD-7 Total Score | 51 | -0.67 | -3.80 to 2.46 |
| Primary Care PTSD Screen Total Score | 51 | 0.08 | -1.01 to 1.16 |
| ABI-R Perpetration Total Score | 50 | -1.31 | -4.06 to 1.43 |
| Controlling Behaviours Scale (Partial) Perpetration Total Score | 50 | -0.17 | -1.05 to 0.71 |
| Propensity for Abusiveness Scale (Anger subscale) total score | 49 | 1.9 | -3.51 to 7.31 |
| Brief self-control scale total score | 50 | -1.49 | -6.41 to 3.43 |
| BIDR-16 total score | 51 | -0.12 | -8.20 to 7.96 |
| *LQ= lower quartile, UQ = upper quartile, sd= standard deviation* | | | |

### **Table S5: Process variables to describe progression criteria**

| **Progression Criteria** | **Process variable** | **Baseline** | | **16 weeks follow-up** | |
| --- | --- | --- | --- | --- | --- |
|  |  | **n** | **median (95% CI)** | **n** | **median** |
| Substance use does not increase in the intervention arm from baseline to 16 weeks follow-up | No. of days of alcohol use in the past 28 days  *Scale Range 0-28* | 104 | 4.0 (1.0 – 12.0) | 22 | 0.5 |
|  | No. of days of heroin use in the past 28 days  *Scale Range 0-28* | 104 | 0.0 (0.0 – 0.0) | 22 | 0.0 |
|  | No. of days of crack use in the past 28 days  *Scale Range 0-28* | 104 | 0.0 (0.0 – 1.0) | 22 | 0.0 |
| Intimate partner abuse does not increase in the intervention arm from baseline to 16 weeks follow-up | ABI-R Perpetration Total Score  *Scale Range 25-125* | 104 | 33.0 (31.0 – 35.0) | 22 | 29.0 |
